# Toward mechanistic endotyping in allergic rhinitis: a State–Place–Time framework for appraising nasal tissue evidence

**DOI:** 10.3389/fimmu.2026.1972016

**Published:** 2026-10-13

**Authors:** Ye Meng, Xiang Gao

**Affiliations:** Department of Otolaryngology–Head and Neck Surgery, Binhai County People’s Hospital, Yancheng, Jiangsu, China

**Keywords:** allergic rhinitis, cellular heterogeneity, mechanistic endotype, mucosal immunity, nasal mucosa, spatial organization, temporal dynamics, tissue state

## Abstract

Allergic rhinitis (AR) heterogeneity is usually described by sensitization, symptom burden, or inflammatory profiles, but these descriptors do not establish which mechanism operates in a particular nasal mucosa. This critical narrative review proposes a State–Place–Time framework that evaluates cellular programs together with their anatomical setting and exposure-related response phase. The framework distinguishes descriptive tissue states from candidate mechanistic modules, measurable mechanistic endotypes, and clinically actionable endotypes. These are operational definitions for this review, rather than a validated classification. Five overlapping examples are examined: IgE–FcϵRI effector activity, late-phase type 2 amplification, barrier–repair dysfunction, separable sensory, vascular, and secretory outputs, and putative stromal maintenance. An author-derived conceptual matrix summarizes human evidence, spatial localization, timing/recurrence, matched perturbation, accessible measurement, and clinical utility; it does not provide an evidence score or patient assignment rule. Human intervention supports IgE-pathway dependence, but this does not establish a validated nasal tissue endotype for treatment selection. Late-phase cytokine responses and epithelial abnormalities are supported by human observations and functional systems, while their stability and predictive utility remain incompletely established. Stromal maintenance remains a hypothesis, with important limits from nasal polyposis sampling and inferred communication. The framework complements existing clinical and inflammatory classifications by identifying missing links and testable alternatives. Progress toward clinical use requires reproducibility within patients, analytical and external validation, and incremental value beyond routine assessment; treatment-selection claims additionally require evidence of differential treatment benefit.

## 1 Introduction

Allergic rhinitis (AR) is readily recognized in clinical practice but remains difficult to explain at the level of the individual nasal mucosa. Sensitization, intermittent or persistent symptoms, disease severity, and disease control remain the basis of diagnosis and treatment (1–3). However, these clinical descriptors mainly capture what patients experience; they do not explain why similar allergen exposure produces different combinations of itch, secretion, obstruction, eosinophilic inflammation, local IgE activity, or treatment response.

The canonical pathway of AR is well established. Aeroallergen exposure induces allergen-specific type 2 inflammation, promotes immunoglobulin E (IgE) production, and increases high-affinity immunoglobulin E receptor (FcεRI) loading on mast cells and basophils. Upon reexposure, histamine, lipid mediators, and other effectors are released within minutes, followed by endothelial activation, interleukin (IL)-5/IL-13-associated inflammation, and eosinophil recruitment over subsequent hours (2, 4, 5). The limitation is not the pathway itself, but its resolution at the tissue level. A linear model does not indicate which cells are primed to respond, where these responses occur within the tissue, or whether a sample represents baseline susceptibility, immediate activation, late-phase inflammation, or treatment-associated changes.

These distinctions have direct clinical relevance. Histamine release near sensory nerve endings may manifest as itch and sneezing, vascular activation contributes to congestion, autonomic glandular responses drive watery secretion, and delayed lymphoid–eosinophil programs may contribute to secondary airflow limitation. Although the mediators involved may overlap, the underlying tissue configurations are not necessarily the same. Describing a patient simply as “type 2-high” may therefore combine biologically distinct processes whose relative contributions vary across anatomical compartments and disease phases.

Human single-cell RNA sequencing (scRNA-seq) studies have identified altered epithelial differentiation and repair programs and heterogeneous immune and stromal states in AR (6, 7). A targeted spatial study reported fibroblast-rich regions and fibroblast–T-cell adjacency, but surgically sampled nasal polyp tissue limits extrapolation to uncomplicated AR (8). These findings locate candidate relationships; they do not establish functional niches or directional mechanisms. Controlled allergen challenge, longitudinal assessment, and patient-derived perturbation systems can test the timing, dependence, and consequences of these observations.

We therefore examine AR heterogeneity across three interdependent tissue dimensions. The first concerns cellular states, in which repair capacity, activation thresholds, differentiation programs, recruitment behavior, and local antibody effector competence may be more informative than cell abundance alone. The second concerns anatomical compartments and candidate functional niches: epithelial, subepithelial, perivascular, gland-associated, and perineural regions constrain which interactions can occur and which outputs can emerge. The third concerns temporal trajectories, in which baseline susceptibility, immediate activation, late recruitment, repeated exposure, and treatment-associated recovery represent different tissue configurations. These dimensions are not orthogonal and do not exhaust all sources of AR heterogeneity; allergen identity, age, sex, season, medication, comorbidity, and systemic immune background modify the tissue state measured at any visit. **Figure 1** summarizes this organizing framework.

**Figure 1:**
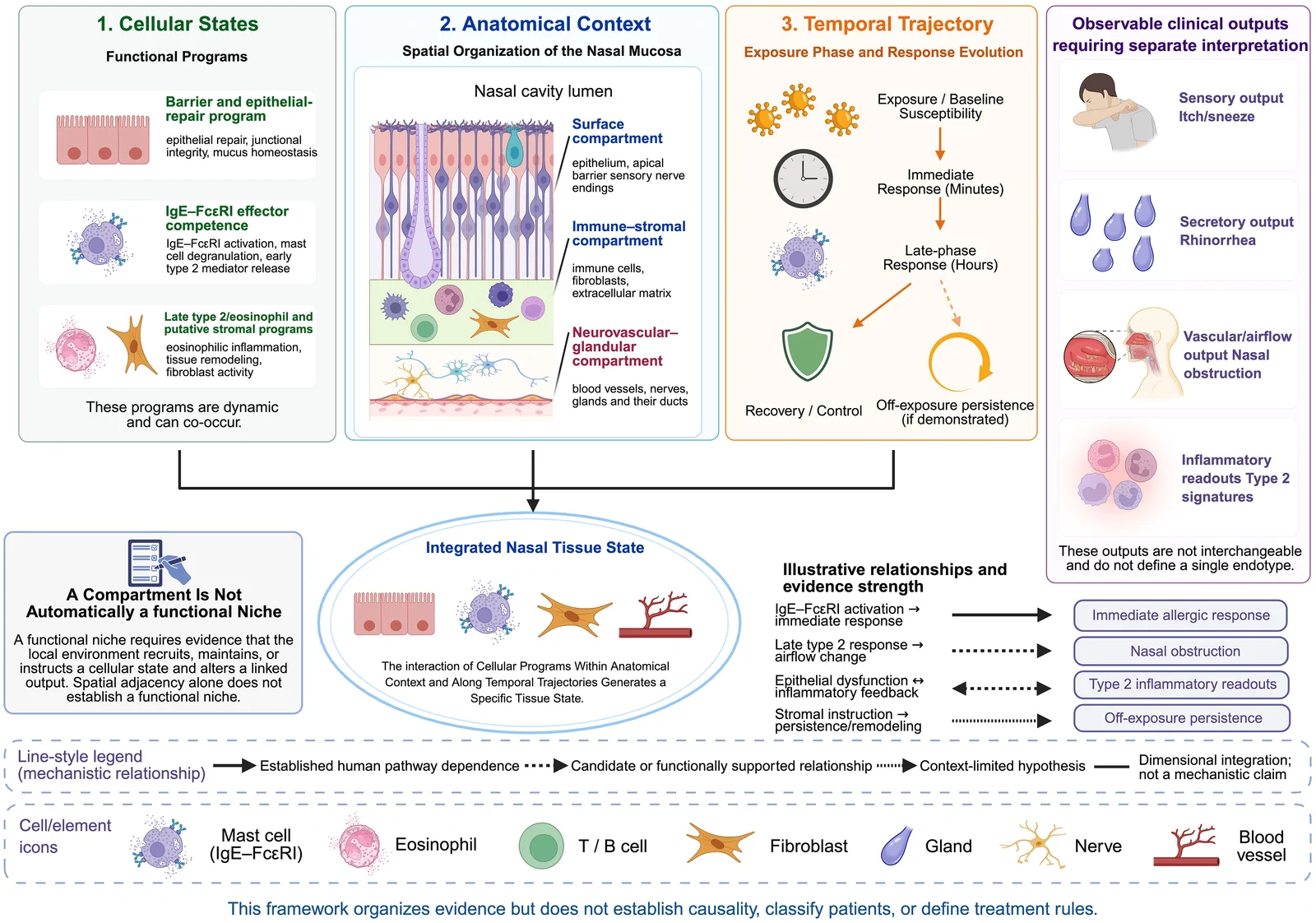
An integrated tissue-state framework for interpreting heterogeneity in allergic rhinitis. The schematic integrates cellular programs, anatomical context, and temporal trajectory without assigning validated patient endotypes. In the illustrative mechanism panel, solid arrows denote established human pathway dependence, dashed arrows denote candidate or functionally supported relationships, and dotted arrows denote context-limited hypotheses. Light-gray connectors denote dimensional integration rather than mechanistic claims. Barrier susceptibility, mediation, and consequence may coexist through reciprocal inflammation-related effects. Nerve-associated, vascular, glandular, and stromal relationships require source- and output-specific testing. Off-exposure persistence is hypothetical unless demonstrated. The output panel separates sensory symptoms, secretion, vascular/airflow effects, and inflammatory readouts. A compartment is not automatically a functional niche: local recruitment, maintenance, or instruction, together with a linked output, must be shown. The framework organizes evidence but does not establish causality, classify patients, or define treatment rules. AR, allergic rhinitis; IgE, immunoglobulin E; FcϵRI, high-affinity IgE receptor. Created in BioRender. qi, L. (2026). https://BioRender.com/a9z6e9b

We treat the nasal mucosa as a dynamic tissue state defined by the cellular programs present, their anatomical setting, and the phase of the response. The review asks when observations made in this state support a biological dependency and when they might inform a patient-level decision. Five domains are examined as worked examples spanning different levels of evidence maturity; they are not proposed as fixed, exhaustive, or equally established AR subtypes. Direct human nasal evidence anchors the analysis, whereas patient-derived systems, related airway diseases, and experimental models are used as supporting context. Genetic studies identify susceptibility factors (9, 10) but do not show which mechanism is active in an individual patient.

## 2 Narrative review approach, evidence appraisal, and operational definitions

This is a critical narrative review. The original search documentation did not retain a reproducible database-specific search strategy or a complete screening flow, and the unsupported August 1 cutoff has therefore been removed. To improve transparency, revision-stage searches of PubMed/MEDLINE, Embase, and Web of Science Core Collection were conducted on 18 September 2026. Five overlapping PubMed queries returned 1,099 records and 823 distinct PubMed identifier (PMID). Embase returned 1,093 bibliographic records and 41 separate trial-registry records. Web of Science returned 977 bibliographic records. PubMed used publication dates from1 January 2016–18 September 2026; Embase and Web of Science used publication years of 2016–2026. No additional language, publication-type, or species restrictions were applied in these retrievals. Across the three bibliographic sets, 2,893 record instances yielded 1,276 unique reports after DOI, PMID, and normalized-title matching. These are retrieval counts, not included-study counts. Metadata-assisted prioritization and focused source appraisal informed purposive additions; complete full-text screening of all retrieved records and dual independent selection are not claimed. Earlier landmark studies were retained through reference checking as context. **Supplementary Methods S3** provides the executed strategies, counts, and selection boundaries; **Supplementary Retrieval Audit S4** consolidates the retrieval summary, query counts, source reconciliation, and focused candidate-appraisal log.

For the targeted update and detailed evidence summaries, primary studies were considered when they addressed well-characterized human nasal tissue or physiology, controlled nasal challenge, longitudinal treatment studies, or a relevant human-derived functional system. Related airway disease and animal experiments were retained only as explicitly indirect context. Studies of systemic biomarkers alone could inform susceptibility or systemic responses but were not treated as nasal mechanism evidence. Reviews and guidelines supplied context and supported reference checking, rather than providing independent primary support. Papers unrelated to nasal biology, nonspecific treatment reports without mechanistic relevance, and unpublished data were outside the detailed appraisal. This purposive narrative selection can miss relevant evidence and does not establish comprehensive coverage. Conference abstracts, preprints, withdrawal/concern flags, and unavailable abstracts were flagged during metadata review rather than automatically counted as eligible primary studies. Trial-registry entries were kept separate and were not used as published efficacy or mechanism results. Narrative additions required a published source, a specific link to a reviewer-identified gap, and an interpretation proportionate to the population, sampling phase, and experiment.

The five domains were selected as worked examples using three criteria: a connection to a defined nasal function or response phase; a human nasal observational or interventional anchor, with disease context stated; and a distinct gap that illustrates progression from description to mechanism or clinical validation. IgE activity illustrates pathway dependence, late type 2 responses illustrate timing, barrier repair illustrates reciprocal causation, physiological outputs illustrate separate target structures, and stromal maintenance illustrates low-maturity inference. They are neither exhaustive nor equally established. Group 2 innate lymphoid cell (ILC2) and dendritic-cell inputs are discussed within type 2 amplification; B-cell/IgE memory within effector competence and recall; complement and inflammasome signals as potentially interacting late-phase programs; and neuroimmune relationships within the distinct sensory, vascular, and secretory domains. A pathway may merit a separate domain if reproducible human localization, selective dependence, and clinically distinct information emerge.

**Figure 2** is an author-derived qualitative appraisal, not a formal evidence-grading system. Its symbols summarize six specified features: human evidence, spatial localization, timing/recurrence, matched perturbation, accessible measurement, and clinical utility. They must not be interpreted as numerical scores, certainty grades, or patient-classification rules. A filled circle denotes substantial support for the specified feature; a triangle denotes partial, emerging, indirect, or context-limited support; and an open circle denotes that the feature was not established in the reviewed evidence. An open circle does not prove biological absence. No symbols are added, weighted, or converted into a total score. **Supplementary Appraisal S2** defines the criteria and rationale for all 30 matrix cells. **Supplementary Table S1** identifies the study design, population, comparator, sample size, sampling method/site, exposure phase, finding, evidence category, and inferential limitation of the representative studies.

**Figure 2:**
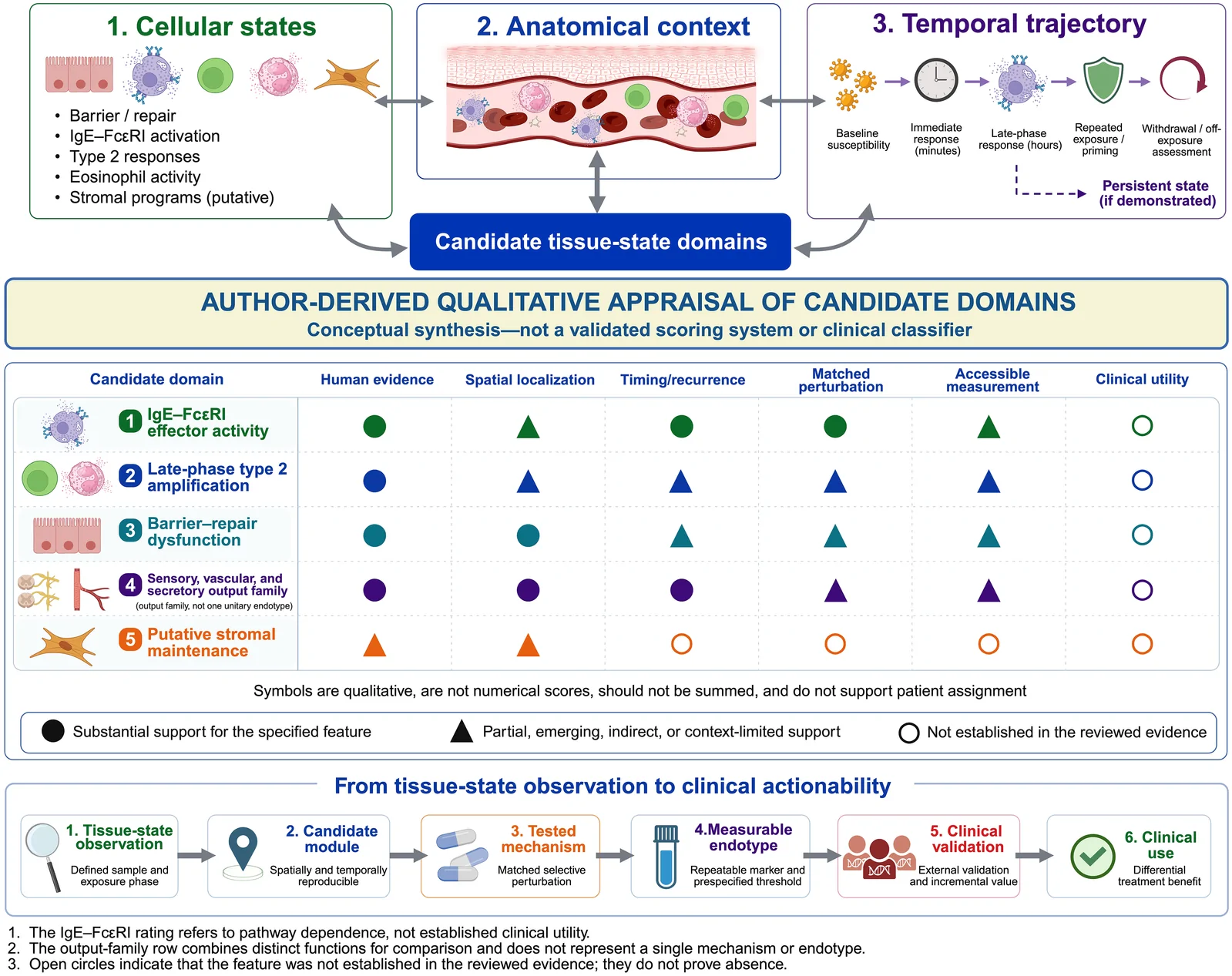
Author-derived qualitative appraisal of candidate tissue domains and their validation requirements. The three tissue dimensions organize observations; the matrix is an author-derived qualitative appraisal of candidate domains, not a validated scoring system or clinical classifier. The six columns assess human evidence, spatial localization, timing/recurrence, matched perturbation, accessible measurement, and clinical utility. Filled circles denote substantial support for the specified feature; triangles denote partial, emerging, indirect, or context-limited support; and open circles denote a feature not established in the reviewed evidence. Open circles do not prove absence. The symbols are qualitative, rather than numerical scores, should not be summed, and do not support patient assignment. **Supplementary Appraisal S2** defines each dimension and explains all 30 cells using evidence sources from **Supplementary Table S1**. The IgE–FcϵRI rating refers to pathway dependence, not established clinical utility. The late-phase row summarizes timed human observations without independently validating stable patient stratification. The output-family row combines distinct functions for comparison and does not represent a single mechanism or endotype. The lower progression defines six requirements: tissue-state observation, candidate module, tested mechanism, measurable endotype, clinical validation, and clinical use. Treatment-selection utility additionally requires differential treatment benefit. Shapes and written labels retain meaning without color. AR, allergic rhinitis; IgE, immunoglobulin E; FcϵRI, high-affinity IgE receptor. Created in BioRender. qi, L. (2026). https://BioRender.com/o4mzxyu

Conflicting or nonconvergent findings are interpreted by comparing disease context, sample source, timing, donor-level replication, and specificity of the perturbation. A result from nasal polyps cannot upgrade direct evidence for uncomplicated AR, and broad anti-inflammatory suppression cannot demonstrate one pathway’s necessity. When these differences leave the proposition unresolved, the appraisal remains partial or unestablished rather than treating positive studies as votes. Failure to detect a rare population is reported as a method- and sampling-dependent negative observation. No formal risk-of-bias score, certainty grade, or independent inter-rater agreement is claimed.

Evidence sources are distinguished throughout the appraisal. Direct human AR evidence comprises nasal measurements or physiology in clinically characterized AR, with asthma comorbidity and comparators identified. Human evidence from chronic rhinosinusitis with nasal polyps (CRSwNP), other airway diseases, or non-AR sampling cohorts is context-limited and indirect for uncomplicated AR. Human-derived explants, primary cells, and cocultures test biological capacity within the reconstructed system. Animal models test experimental mechanisms but do not establish patient-level nasal dependence. Computational predictions and spatial adjacency nominate relationships without establishing binding, direction, necessity, or local instruction. A study can supply more than one evidence category; each experiment is interpreted separately. Serial challenge supports observed temporal order, whereas a mechanism-matched human intervention provides stronger pathway-dependence evidence.

Terminology was calibrated to evidential strength. “Observed” and “associated with” refer to direct measurements; “can” is used when biological capacity is supported by patient-derived functional experiments; “compatible with” and “nominates” describe computational or spatial inference; and “depends on” is reserved for appropriately matched perturbation evidence. “Compartment” denotes an anatomical region, “neighborhood” denotes a reproducible cellular arrangement, and “functional niche” is used only when the local environment is shown to recruit, maintain, or instruct a cellular state and to alter a linked output.

The following nested terms are operational definitions proposed for this review, not universally accepted diagnostic categories. A tissue state describes measured cellular programs, location, and phase. A candidate mechanistic module proposes a biological dependency; a demonstrated module requires a perturbation that tests that dependency in the relevant system and phase. A measurable mechanistic endotype additionally requires a patient-level marker or surrogate with analytical validity and reproducibility under a defined exposure condition. Repeated assessment within the same patient must distinguish a stable dependency or reproducible inducible trajectory from an isolated exposure episode and technical noise. A clinically actionable endotype further requires external validation and incremental utility for a prespecified decision beyond routine assessment. Diagnostic, prognostic, and treatment-selection uses require different forms of validation: only the last specifically requires differential treatment benefit, preferably demonstrated through a validated marker-by-treatment interaction. **Figure 2** illustrates this progression without implying that any of the five domains has completed it.

## 3 What is being measured when AR appears heterogeneous?

### 3.1 Clinical variation is an output, not a mechanism

Sneezing, itch, rhinorrhea, and obstruction arise from different combinations of sensory activation, glandular secretion, plasma exudation, vascular engorgement, and inflammatory recruitment. Summing them into a total symptom score is useful for trials but obscures their anatomical origin. The same applies to familiar inflammatory labels. “Eosinophilic AR” may reflect strong recruitment, prolonged tissue survival, or delayed clearance; “local IgE” may represent mucosal production or delivery through plasma exudation. Observable phenotypes become mechanistically informative only when linked to the process that generates them.

This distinction is especially important at disease boundaries. Healthy controls, symptomatic nonallergic rhinitis, systemically sensitized AR, and local allergic rhinitis are different comparators. Local allergic rhinitis demonstrates the problem clearly: a reproducible nasal allergen response can occur despite negative skin tests and serum allergen-specific IgE (11, 12). Conversely, systemic sensitization does not show that a given allergen is driving current nasal symptoms. Childhood onset, asthma comorbidity, and polysensitization further alter the biological background (13).

### 3.2 Every specimen is a partial view of the tissue

Anatomical site and sampling method determine which form of heterogeneity is visible. Brushing favors surface epithelium; curettage captures epithelium and limited inflammatory material; lavage and absorptive matrices collect released products; biopsy preserves deeper architecture but covers a small field. Paired single-cell analysis has shown that brushing underrepresents fibroblasts, endothelial cells, B cells, and glandular populations relative to tissue (14). That study was not confined to AR, so its quantitative estimates cannot be transferred directly; however, the sampling principle is nonetheless robust.

Tissue dissociation can lose granulocytes, fragile epithelial cells, and stromal elements, while *ex vivo* stress may introduce transcriptional states. Regardless of the number of cells profiled, the number of independent biological replicates is determined by the number of donors. Cell-state claims require donor-aware analysis and, when position matters, confirmation by protein imaging or targeted spatial methods.

### 3.3 Heterogeneity is represented within a multidimensional tissue-state framework

At any clinical visit, the nasal mucosa can be described as a tissue state that combines cellular programs, anatomical context, and response phase. Patients with similar symptom scores may occupy different biological states, and the state of a single patient may change after allergen exposure or treatment. This representation is descriptive: it records what was measured, where it was measured, and when it was measured. Allergen, season, age, medication, and comorbidity influence the observed state but are not separate additional axes of the model (**Figure 1**).

Exposure and treatment history are therefore part of the interpretation of every specimen. A sample obtained during the pollen season may reflect recent exposure, cumulative inflammation, and medication effects as much as baseline susceptibility. Programs that appear only during active exposure or disappear after treatment are better regarded as context-dependent states unless longitudinal evidence shows that they recur or persist within the same patient.

Age and exposure context are modifiers of all three dimensions. A pediatric birth cohort identified distinct age-of-onset trajectories in 1,050 children. Its blood-transcriptomic analysis used a separate 36-child subset: 12 early-onset, 12 late-onset, and 12 never/infrequent AR, sampled at mean ages of 6.58, 6.75, and 6.92 years, respectively (13). This systemic subset does not constitute a validated nasal tissue classification. Adult challenge-derived thresholds cannot therefore be assumed to transfer to children. Developmental stage, age-appropriate sampling feasibility, and age-stratified external validation are needed; sex and relevant hormonal context should likewise be recorded rather than assigned an unproven mechanistic effect. Allergen identity, seasonal versus perennial exposure, recent exposure intensity, infection, irritants, asthma, and medication can change the measured baseline. Studies should define whether baseline means prechallenge, out of season, low exposure, or postwashout, and document safety-appropriate medication intervals. Comparisons should match or adjust for these factors and test prespecified effect modification when sample size permits. A prechallenge measurement during active perennial exposure is not necessarily an unexposed state.

Direct nasal microbiome observations also illustrate context dependence. Out-of-season ragweed challenge in 19 allergic participants and 12 controls found no significant acute effect on overall microbial diversity despite clinical responses, while microbial community composition differed by sex and *Staphylococcus aureus* carriage (15). This does not establish causal sex effects or exclude finer microbial changes. It shows why baseline host factors should be recorded and why a clinical response does not require every measured tissue feature to change.

## 4 Cell states: the first tissue dimension of AR heterogeneity

Cell atlases become useful when they reveal a change in function that cannot be inferred from lineage counts. In AR, epithelial repair, immediate effector competence, eosinophil trafficking, lymphoid cytokine production, and local antibody recall are informative because each can alter a defined phase of the response. Their interpretation nevertheless depends on place and time: an IL-13-responsive epithelial cell during late challenge has a different meaning from the same transcript at an untreated seasonal baseline, and a circulating eosinophil state does not become a nasal mechanism until tissue entry has been shown.

### 4.1 Epithelial state defines the response threshold

Basal-cell expansion can reflect repair, stalled differentiation, or replacement of damaged luminal cells. Human profiling with protein and primary-cell validation identified an allergy-associated basal state with impaired barrier-forming capacity (6). A current study recruited 49 participants: nasal mucosal scRNA-seq included 25 with AR and 14 with nonallergic rhinitis, scATAC-seq used a subset of 16 participants and eight with nonallergic rhinitis, and an additional cohort of five participants with AR and five with nonallergic rhinitis underwent immunofluorescence validation (7). The symptomatic comparator helps identify disease-associated epithelial and stromal programs, but cross-sectional analyses of differentiation and communication do not establish causal direction.

In house dust mite (HDM)-associated AR, nasal secretions and inflammatory mediators can impair barrier properties in primary epithelial cultures (16). Experimental barrier disruption increases allergen passage in human cells, while sensitization and mast-cell effects in that study were mainly tested in mice (17). Exposure-chamber observations link preserved epithelial programs to resistance to clinical disease, without establishing whether the barrier is cause or consequence (18). Progenitor memory in chronic type 2 sinonasal disease and the cross-organ epithelial barrier hypothesis provide supporting context, rather than proof of a stable presensitization defect in uncomplicated AR (19–21).

A 2026 study of 30 AR participants and 30 controls associated epithelial S100A8 with reduced junction-associated proteins. S100A8 silencing restored those proteins in HDM-stimulated human nasal epithelial cells (22). This finding combines human association with cellular perturbation; it does not establish durable barrier restoration or clinical benefit in patients. Another 2026 primary study links basal-cell inflammatory responses and mast-cell mediators to impaired epithelial progenitor function in human-derived assays (23). Together, these findings support reciprocal barrier–inflammation interactions as a testable mechanism. Antecedent susceptibility, mediation, and consequence can coexist and reinforce one another; however, sustained feedback after exposure withdrawal in the intact AR nose still requires direct testing.

Differentiated states provide a second readout: loss of mature ciliated or club-like programs differs from an IL-13-induced mucus program. Serial sampling before the season, during exposure, and after washout is needed to distinguish a transient cytokine imprint from impaired differentiation.

Rare epithelial sensors may alter response thresholds despite low abundance. A 2026 HDM-AR study found increased solitary chemosensory cell (SCC), IL-25, and protease-activated receptor 2 (PAR-2) signals in nasal swabs or secretions (24). Its biopsy arm included CRSwNP with and without AR, and its culture systems were partly polyp-derived. PAR-2 perturbation therefore supports cellular capacity within those systems rather than an obligatory circuit in uncomplicated AR. A separate multimodal study found little support for a substantial nasal neuroendocrine-cell population, which limits transfer of the pulmonary model without proving that every rare nasal sensor is absent (25).

Epithelial cytokines should not be collapsed into an “alarmin-high” label. Thymic stromal lymphopoietin (TSLP) signaling requires cytokine receptor-like factor 2 (CRLF2) together with IL-7Rα (26). Blood-derived ILC2 cultures distinguish TSLP-associated survival from stronger IL-33 activation, without identifying a source–recipient circuit in intact AR mucosa (27). In CATNIP, tezepelumab plus cat subcutaneous immunotherapy (SCIT) improved challenge responses during treatment, but the primary symptom endpoint at 1 year after withdrawal was not superior to SCIT alone (28). This demonstrates pathway engagement in that trial context, not a validated TSLP-dominant endotype.

Barrier-related hypotheses require different tests. A susceptibility claim requires detection before the relevant response and prediction after accounting for recent inflammation. A mediation claim requires selective barrier restoration to reduce allergen passage or a linked downstream response. A maintenance claim requires persistence or recurrent functional feedback after exposure withdrawal. These roles can coexist. A signature that changes with inflammation may be a consequence or a pharmacodynamic marker, yet this alone neither proves nor excludes a contributory feedback mechanism.

### 4.2 Immediate effector competence is more than sensitization

Mast cells and basophils share IgE–FcϵRI signaling but occupy different temporal and anatomical roles. Resident mast cells can respond within minutes; basophils enter from blood and may contribute later. Anti-IgE treatment reduces free IgE, lowers FcϵRI expression, and attenuates nasal challenge responses, providing the strongest human evidence for a pathway-level dependency in AR (29, 30). Serum-specific IgE alone is less informative because it does not report local antibody availability, receptor loading, cell position, or activation threshold.

Mast-cell specialization remains incompletely mapped in uncomplicated AR. Tryptase/chymase states and transforming growth factor β (TGF-β)-associated differentiation in nasal polyposis tissue illustrate local environmental capacity, but cannot be used to construct an uncomplicated AR mast-cell atlas without direct validation (31).

Human intervention and minute-scale physiology support IgE-pathway dependence (29, 30). Position-specific hypotheses require additional evidence: a mast cell near a sensory ending or venous structure is anatomically capable of influencing those outputs, but adjacency alone does not establish a directional mast-cell–nerve or mast-cell–vascular relationship in AR. Neither total mast cell counts nor serum sensitization identifies which local dependency determines residual symptoms or differential treatment benefit.

### 4.3 Eosinophilia contains several biologically distinct steps

An eosinophil count combines production, circulating priming, tissue entry, survival, and degranulation. A single-cell study identified a TNF-responsive state in circulating eosinophils from AR participants (32). Timed nasal challenge links delayed type 2 inflammation and eosinophil-associated programs to late obstruction (33, 34). In a small single-blind sequential study of placebo and corticosteroid pretreatment involving 10 participants with AR and mild asthma, corticosteroid pretreatment reduced mature nasal eosinophils and activated ILC2s after challenge, while eosinophil progenitors increased (35). These observations support different stages of a blood-to-tissue trajectory. They do not trace the circulating TNF-responsive state to a specific nasal descendant.

Activation is a further dimension. Granule proteins in lining fluid may reflect regulated secretion, cytolysis, or sampling trauma, while a low intact-cell count after cytolysis can underestimate earlier tissue accumulation. Neither finding can be interpreted without timing: endothelial entry markers should precede accumulation, and accumulation should precede or coincide with release products if the proposed sequence is correct.

Eosinophils localize near nasal nerves after allergen challenge before increased reflex secretion is observed (36). This sequence is compatible with neural sensitization, but neural recruitment, eosinophil effects, and a shared upstream signal remain alternatives. Selective eosinophil perturbation with a later reflex endpoint could distinguish these explanations; proximity alone cannot establish the circuit.

### 4.4 Lymphoid source and local immune memory

ILC2s and dendritic cells can contribute to amplification and antigen presentation without defining mutually exclusive patient categories. Identifying an IL-13 source requires source-resolved measurements; assigning an ILC2-dependent endotype would additionally require a selective perturbation or validated predictive marker beyond shared cytokine levels (27, 35, 37).

Allergen-specific CD4 T cells provide specificity, but phenotype alone does not establish nasal function. The cytokine-rich Th2A-like state was defined mainly in blood and nonnasal allergic tissues (38), and T follicular- or peripheral-helper programs are only plausible sources of local B-cell help (39). In AR, each claim requires allergen specificity, mucosal localization, and proximity to the proposed recipient.

Off-season nasal explants released IL-5 after relevant allergen or Bet v 1 peptide stimulation, supporting local recall (40). The system excludes new cellular recruitment but does not establish canonical tissue residence. Allergen immunotherapy (AIT) studies show coordinated molecular changes, while causal separation of resident and circulating Th2 memory comes mainly from mouse airway studies and review synthesis (41–44). Paired blood–nasal T-cell receptor (TCR) tracking with off-season localization can distinguish persistent clones from repeated recruitment.

ILC2s can supply IL-5 and IL-13 without antigen recognition. Challenge-associated nasal ILC2 activation falls with corticosteroid pretreatment, but the small human intervention cohort included participants with mild asthma (35). AR-derived culture experiments and neuromedin U (NMU)-associated responses support biological capacity; intact-system selective dependence remains mainly experimental (45–47). Human association, human-derived stimulation, and mouse perturbation in these studies must be interpreted separately.

The source of a shared cytokine cannot be inferred from the cytokine itself. A nasal IL-13-high response may arise from Th2 cells, ILC2s, or both. Source resolution becomes clinically relevant only if it changes intervention. Until an ILC2-selective input predicts outcome beyond nasal IL-13 and cytology, “ILC2-dominant AR” remains a research hypothesis.

Nasal B-lineage states may contribute to local recall. IgE detection alone does not distinguish mucosal synthesis from recruitment or plasma exudation. A small local-AR challenge study found plasmablasts and recombination signals compatible with sequential IgG-to-IgE switching (11). AIT studies link memory clones to IgE responses, while peripheral CD38-positive B-cell associations and mechanistic mouse experiments address different levels of evidence (48–50). Early SCIT sampling showed divergent nasal, blood, and lymph-node antibody changes (51). These compartment-specific findings do not establish where durable nasal IgE memory resides in untreated individuals.

A surgical study recruited 30 HDM-AR participants with septal deviation and 21 septal-deviation controls. IgE-positive plasma-cell observations and tissue IgE were associated with endoplasmic reticulum stress markers; 4-phenylbutyric acid reduced IgE in AR explant assays using six samples (52). This supports local tissue production capacity and stress-associated regulation. Broad tissue-level inhibition does not isolate B-lineage necessity, distinguish a durable memory reservoir, or demonstrate clinical benefit. The effect was observed at 12 and 24 h, with no reduction at 48 h; durability cannot be assumed.

### 4.5 Mixed programs are modifiers until proven otherwise

Type 2 inflammation in the nose coexists with complement, inflammasome, interferon, IL-17, and neutrophil-associated programs. Serial challenge has detected complement and IL-1β alongside IL-5 and IL-13 (33); IL-17A can enhance IL-13-induced C-C motif chemokine ligand 26 (CCL26) production in patient-derived epithelium (53); and TLR7/8 stimulation elicits interferon-associated responses (54). These interactions may change the magnitude or duration of disease without defining a separate mechanism.

The same restraint applies to dendritic cells and macrophages. Human challenge supports accumulation of a historical dendritic-cell phenotype (37), whereas directional neuron-to-dendritic-cell signaling was shown in mouse skin (55). Patient biopsies associate macrophages and NLRP3-related staining with AR, but selective inhibition and disease attenuation were tested in cells and an OVA mouse model (56). Retrospective eosinophil-, neutrophil-, and cytokine-based clusters (57) also remain vulnerable to infection, irritants, medication, and sampling time. Such programs are contextual modifiers until they recur, are shown to be mechanistically distinct, and predict treatment response.

Cell-state studies in AR identify recurring programs related to epithelial repair, immediate effector activity, eosinophil recruitment, and local immune recall. Their main contribution is to define functions that may differ between patients and across response phases. Whether these programs influence symptoms depends in part on their position within the tissue, which makes anatomical context the next level of analysis.

## 5 Anatomical compartments and candidate functional niches: the second tissue dimension

Spatial evidence in AR is less mature than the evidence for IgE-dependent activation or epithelial dysfunction. Its immediate contribution is not a validated atlas of functional nasal niches, but a change in the unit of interpretation: a mediator acquires biological meaning through the compartment in which it is produced and the structures it can reach. The discussion below therefore distinguishes anatomical compartments, observed cellular neighborhoods, and functional niches. Only the last term implies local instruction or maintenance demonstrated by more than proximity.

### 5.1 Anatomical compartments constrain symptom generation

“Nasal mucosa” is not a single compartment. Mechanistic interpretation should distinguish at least the luminal epithelium, epithelial–stromal boundary, superficial lamina propria, venous/perivascular regions, gland-associated stroma, and nerve-associated regions. These compartments differ in access to allergen, blood, and neural signals. Barrier failure acts at the surface; itch and sneeze require sensory access; congestion depends strongly on capacitance-vessel engorgement and permeability; and watery secretion may be glandular, exudative, or both. The same mediator can therefore have different consequences depending on the target structures accessible at the relevant time.

Sampling method is consequently part of the biological result. Brushing supports repeated epithelial measurements but underrepresents fibroblasts, endothelium, glands, and many B-lineage cells relative to tissue (14). Lavage measures released products while obscuring cellular source and introducing dilution; plasma exudation also introduces blood proteins. Biopsy preserves architecture but samples a small field. Spatial claims require a defined site and orientation, gland and vessel context, and donor-aware—not field-level—replication.

### 5.2 The epithelial–subepithelial signaling boundary

The epithelial–subepithelial interface is the first candidate organizing boundary. Epithelial TSLP, IL-33, and IL-25 may signal to adjacent immune cells, but coexpression in separate clusters establishes only receptor compatibility. Spatial proximity, a bioactive mediator, the complete receptor complex, and downstream activation in the recipient are separate requirements.

For example, TSLP signaling requires CRLF2 together with IL-7Rα, making an inferred TSLP–IL7R edge incomplete (26). IL-33 must be released or processed rather than merely present in epithelial nuclei, and an SCC-derived IL-25 signal requires a correctly identified rare source besides IL-17RB-positive recipients (24). Culture establishes capacity and therapeutic perturbation establishes pathway modulation; neither demonstrates the source–recipient relation in AR tissue.

The basement membrane is also an active filter. Its composition and thickness alter migration and mediator diffusion; basal cells and fibroblasts on opposite sides may participate in reciprocal repair signaling. An epithelial IL-13 response can induce CCL26 in patient-derived cells (53), and CCR3-positive eosinophils are positioned to respond. This establishes compatible components, not a closed feedback loop. The loop would require epithelial activation before eosinophil entry, spatially matched ligand and receptor proteins, and loss of recruitment after direction-specific blockade. If both compartments change simultaneously with a shared IL-13 exposure, reciprocal instruction has not been shown.

### 5.3 Fibroblast–immune neighborhoods and the unresolved question of stromal instruction

Fibroblast states can influence chemokine production, extracellular matrix organization, tissue remodeling, and growth-factor availability, but their interpretation depends on anatomical localization and functional context rather than fibroblast abundance alone.

The 2026 Xenium study reports fibroblast-associated spatial patterns and candidate ligand–receptor signals (8). Both surgical groups had nasal polyposis; its abstract reports 10 participants per group, while the recruitment methods report nine per group. Neither count is treated as definitive. These observations are context-limited for uncomplicated AR, and inferred communication cannot establish stromal instruction. Replication and selective functional testing are needed before a maintenance mechanism is claimed.

Independent AR/nonallergic-rhinitis multiomics studies identify inflammatory and matrix-associated fibroblast states (7). Communication and trajectory analyses remain inferential. These studies nominate stromal hypotheses but do not demonstrate a fibroblast-maintained niche or clinically identifiable stromal endotype.

### 5.4 Perivascular entry, congestion, and retention

The nasal vasculature both causes obstruction through vascular engorgement and increased permeability and provides the entry route for recruited leukocytes. Immediate mediator-driven flow changes precede endothelial adhesion and chemokine-guided migration. Candidate axes such as VCAM-1–VLA-4, CCL11/CCL26–CCR3 and CCL17/CCL22–CCR4 become trafficking mechanisms only when endothelial protein display and effects on leukocyte entry are demonstrated; transcript compatibility is insufficient.

Pericytes, vascular smooth-muscle-like cells, and perivascular fibroblasts are poorly recovered by dissociation, and broad endothelial clusters can mix arterial, venous, capillary, and lymphatic states. Given that congestion and leukocyte entry are primarily vascular questions, vessel morphology and venous/sinusoidal localization matter more than further subdivision of an anatomically unresolved cluster.

Classic unilateral challenge experiments separated local albumin-rich plasma exudation from contralateral reflex glandular secretion, demonstrating that vascular leakage and neural secretion are different outputs of the same exposure (58). This physiological resolution has largely been lost in contemporary omics studies. Serial airflow, exudation markers, and source-resolved secretion can determine whether early vascular changes or later cellular inflammation accompanies obstruction; fixed anatomy and persistent obstruction despite anti-inflammatory perturbation would falsify an inflammatory assignment. Spatial endothelial profiling is useful only after the physiological output has been defined.

### 5.5 Neuroimmune, neurovascular, and gland-associated compartments and physiological dependencies

Neural biology separates into afferent sensation, local axon-reflex signaling, and autonomic control of glands and vessels. Histamine-sensitive sensory activation, parasympathetic secretion, and neurovascular tone can amplify symptoms without a proportional increase in inflammatory-cell burden; they should not be collapsed into one pathway.

Evidence differs across neural circuits. Histamine-sensitive physiology is supported in humans, whereas directional substance P–dendritic-cell and nociceptor–B-cell findings arise mainly from mouse skin or airway studies (55, 59). Nasal polyposis mast-cell differentiation, AR-derived substance P responses, and NMU-related cellular observations address separate experimental contexts (31, 47, 60). They do not reconstruct a one-directional neuroimmune circuit in uncomplicated AR.

An open-label randomized-order crossover challenge in 25 persistent-AR participants and 16 controls found different nasal response patterns to leukotriene D4 and histamine: resistance effects differed from effects on sneezing and discharge (61). This directly supports separable human physiological outputs. Exogenous mediator administration does not establish which endogenous cellular circuit is necessary, nor does it validate a marker-guided choice between therapies.

Human AR evidence is strongest for systems physiology and temporal anatomy: allergen evokes neural reflex secretion distinct from ipsilateral plasma exudation, and eosinophils accumulate near nerves before heightened reflex responsiveness (36, 58). By contrast, a major population of nasal neuroendocrine cells has not been confirmed (25). The negative evidence favors direct neural fibers and their immune partners over an extrapolated pulmonary neuroendocrine-cell model.

The phrase “neuroimmune unit” is useful only when separated into nerve–mast-cell, nerve–eosinophil, nerve–ILC2, neurovascular, and nerve–gland relationships. Histamine-sensitive itch, cholinergic watery secretion, and vascular obstruction have different effectors and need not covary. Selective H1 blockade, anticholinergic treatment, and objective measures of vascular responses or exudation already provide output-specific perturbations. A composite molecular “neural” score should not be interpreted as an endotype unless it adds information beyond these simpler tests.

### 5.6 When can a compartment or neighborhood be called a functional niche?

Current spatial studies are most useful for locating candidate interactions and distinguishing processes arising at epithelial, vascular, glandular, neural, or stromal sites. They provide a map of where a mechanism could operate, but functional direction still requires targeted experiments or human perturbation. Detailed evidence for individual anatomical relationships is considered above; the broader question is how these relationships change across the exposure–response cycle.

## 6 Temporal trajectories: the third tissue dimension of AR heterogeneity

A nasal sample is inseparable from its position on the exposure–response curve. Baseline, immediate reaction, late inflammation, and posttreatment recovery represent distinct cellular states and provide different causal information. Treating these time points as replicates of a stable endotype is a major source of apparent inconsistency between studies.

### 6.1 Baseline records response potential

At baseline in sensitized individuals, allergen-specific memory, IgE-loaded mast cells, epithelial susceptibility, and tissue architecture establish response potential. A normal-looking baseline sample does not exclude a large inducible response, and an inflammatory baseline sample may reflect recent natural exposure. The value of a baseline biomarker lies in what it predicts on challenge or during the next exposure period.

### 6.2 Immediate and late responses are different tissue configurations

Within minutes of exposure, IgE cross-linking activates prepositioned mast cells and sensory pathways and triggers plasma leakage and glandular secretion. Recruitment is too slow to explain this phase. A marker assigned to immediate disease must therefore be present before or within minutes of challenge and must track an immediate output; a leukocyte population appearing hours later cannot explain the first sneeze or early plasma leakage. A late-phase response represents a temporally defined exposure-induced state and should not be interpreted as chronic disease without evidence of persistence after stimulus withdrawal.

Hours after challenge, cytokines and recruited cells form a different tissue configuration. Grass-pollen studies detect type 2, inflammasome-related, and complement-associated changes (33). In a birch-pollen trial, eight of 20 challenged participants showed high nasal IL-13 at 2–8 h and secondary airflow reduction (34). Challenge occurred on three consecutive days, but independent test–retest classification, an externally validated threshold, and a marker-by-treatment interaction were not established. The causal chain remains incomplete. Lack of reproducible status would limit stable classification; absence of a treatment interaction would specifically prevent treatment-selection use, while leaving possible explanatory or pharmacodynamic value.

### 6.3 Repeated exposure can mimic stable disease

Repeated exposure lowers the response threshold, increases eosinophilic inflammation, and prolongs symptoms. Controlled repetitive challenge produced clinical priming and Th2 inflammation without clear structural remodeling (62). Persistent symptoms may thus reflect repeated inducible responses without a fixed tissue lesion.

Several substrates could sustain recurrence: allergen-specific lymphocyte clones, IgE-loaded mast cells, memory B cells capable of regenerating IgE, or epithelial and stromal programs that outlast active inflammation. Adaptive recall is established. Durable epithelial or fibroblast memory in uncomplicated AR is not. It would require persistence after withdrawal of the stimulus and an altered response on rechallenge, preferably supported by chromatin or lineage evidence.

### 6.4 Treatment reveals mechanism only when timing is explicit

Treatments interrogate different response phases. H1 blockade mainly affects selected immediate outputs, corticosteroids suppress several late inflammatory programs, anticholinergics reduce glandular secretion, anti-IgE lowers effector competence, and allergen immunotherapy modifies immune recall (63, 64). Phase-selective change is more informative than broad suppression for identifying a biological dependency. During grass-pollen immunotherapy, nasal epithelial and inflammatory signatures changed with treatment (41), whereas 2 years of sublingual immunotherapy did not meet the predefined criterion for a durable third-year outcome (65). These findings demonstrate biological engagement but do not by themselves identify pretreatment responders or establish durable tissue reprogramming.

## 7 Integrating the three dimensions: from tissue states to candidate endotypes

### 7.1 A tissue state is not yet a mechanistic module or endotype

The candidate tissue-state framework organizes observations according to cellular program, anatomical context, and response phase. These dimensions clarify what was measured, but they do not by themselves establish a biological dependency. We therefore compared candidate domains in terms of human evidence, spatial localization, timing/recurrence, matched perturbation, accessible measurement, and clinical utility (**Figure 2**). The five domains are worked examples that differ in evidence maturity; they are not intended as an exhaustive or mutually exclusive classification of AR.

Mechanistic contributions may overlap and may change across the course of a response. IgE-dependent initiation, late-phase type 2 inflammation, epithelial susceptibility, along with sensory, vascular, or glandular outputs can coexist within the same patient. Their relative importance may also change after treatment, making the mechanism responsible for residual symptoms more informative than assignment to a fixed subtype.

### 7.2 Candidate domains differ in evidential closure

IgE–FcϵRI activity has strong pathway-level support from human temporal observations and anti-IgE intervention (29, 30). The relevant proposition is local effector competence, not sensitization alone. Local AR illustrates the limits of serum IgE as a surrogate (11, 12). A validated nasal tissue treatment-selection endotype would additionally require a reproducible tissue-proximal marker that improves selection beyond routine history, sensitization testing, and challenge. Pathway dependence and clinical marker utility are distinct claims.

Late-phase IL-5/IL-13 responses and secondary airflow changes define a timed exposure-associated pattern (33, 34). Small cohorts and incompletely validated repeatability of classification limit patient stratification. Reproducibility, threshold validation, and external outcome testing are needed. Evidence of differential treatment benefit is required for treatment selection but not for every possible explanatory or diagnostic use.

Barrier–repair dysfunction has convergent anatomical and human-derived functional support (6, 7, 16, 22, 23). Inflammation can both disrupt the barrier and be amplified by altered epithelial responses. Direction, persistence, and clinical utility therefore require separate tests. A selective epithelial perturbation should change a linked output rather than simply accompany broad inflammatory suppression. A brushing-based panel would require demonstrated incremental predictive or treatment-selection value before clinical implementation.

Sensory, vascular, and secretory outputs should not be interpreted as a single mechanistic domain. Histamine-sensitive sensory activation, cholinergic glandular secretion, plasma exudation, and capacitance-vessel engorgement represent separable dependencies with different anatomy and perturbations. This domain is useful for organizing residual symptoms, but clinically meaningful stratification should occur at the level of specific outputs. A composite score that does not outperform selective physiology or treatment response would add complexity without clarifying dependence.

Putative stromal maintenance is included as a low-maturity boundary case, not as a mechanism supported to the same degree as IgE–FcϵRI effector activity. Available spatial and multiomic studies identify fibroblast-associated states and opportunities for stromal–immune interaction, but both are limited by disease context, comparator selection, or computational inference (7, 8). Neither study shows that fibroblasts maintain inflammation independently of continuing exposure, determine treatment response, or represent a clinically identifiable patient subgroup. Replication in uncomplicated AR, persistence after allergen withdrawal, and direction-specific stromal perturbation would be required before this domain could be considered a mechanistic module.

**Figure 2** provides a conceptual summary of the reviewed evidence, not a reproducible certainty ranking or clinical score. **Supplementary Appraisal S2** explains each symbol; **Table 1** summarizes present interpretations, evidence sources, and decisive next tests. Changes in a symbol do not assign or reassign a patient to an endotype.

**Table 1:** Current interpretation and decisive next tests of candidate tissue domains in allergic rhinitis.

Candidate domain	Current status and clinical interpretation	Decisive next test or falsifying result	Evidence source and disease context
IgE–FcϵRI effector activity	Established pathway-level mechanistic module but not yet a completed tissue-proximal treatment-selection endotype.	A tissue-proximal marker must add predictive or treatment-selection value beyond history and routine sensitization testing. Absence of incremental value would limit it to mechanism confirmation.	Direct human AR challenge and anti-IgE trials; local-AR observations (11, 29, 30).
Late-phase type 2 amplification	Timed response phenotype and candidate mechanistic module; patient-level stability remains uncertain.	Independent within-patient recurrence, threshold validation, and external outcome validation; differential benefit for treatment selection. Unstable classification prevents a stable label, while absence of interaction prevents treatment-selection use.	Direct human AR challenge; small cohorts, with timed fluid/physiologic observations (33, 34).
Barrier–repair dysfunction	Reproducible epithelial tissue state and candidate susceptibility or maintenance module; causal direction remains unresolved.	Test susceptibility, mediation, and maintenance separately; these roles can coexist. Selective barrier perturbation must change a linked response. Parallel improvement after broad suppression does not resolve the direction.	AR tissue and human-derived assays; some experiments or comparator systems are indirect (6, 7, 16, 22, 23).
Sensory, vascular, and secretory output family	A family of separable physiological dependencies rather than a single mechanistic endotype.	Output-specific measurements and selective perturbations must identify the dominant dependency. A composite signature that adds no value beyond simpler physiological assessment would not justify molecular endotyping.	Direct AR physiology and timed biopsy observations; proposed cell circuits also rely on indirect/model-based evidence (36, 47, 58, 60).
Putative stromal maintenance	State–place hypothesis; neither a demonstrated functional niche nor an actionable endotype.	Requires replication in uncomplicated AR, demonstration of off-exposure persistence, and direction-specific stromal perturbation. Disappearance with general inflammatory control would favor interpretation as a secondary disease record rather than a driver.	AR/NAR multiomics and nasal-polyposis spatial tissue; communication remains inferred (7, 8).

*AR*, allergic rhinitis; *IgE*, immunoglobulin E; *FcϵRI*, high-affinity IgE receptor.

### 7.3 Accessible measurement separates a mechanistic module from a measurable endotype

Diagnostic, pharmacodynamic, prognostic, and treatment-selection biomarkers answer different questions (66). A cytokine decrease during AIT may demonstrate a biological response without predicting who benefits. Treatment selection requires evidence of differential benefit, preferably a prespecified marker-by-treatment interaction in a randomized comparison. Correlation with improvement in one treatment arm is insufficient. Assay development must define the tested population, specimen, exposure phase, comparator, and intended action.

Local allergen-specific IgE illustrates the point. In 60 HDM-AR patients and 143 healthy controls, local Der p/Der f IgE correlated with nasal type 2 cytokines more strongly than serum IgE (67), showing compartmental concordance but not incremental value in uncertain cases. A meta-analysis found limited sensitivity for nasal-specific IgE and basophil activation compared with provocation testing (68); a positive result can support local AR, but a negative one cannot exclude it.

An accessible patient-level marker is a feasible and repeatable measurement in the intended setting, not simply a technically measurable signal. Blood testing is comparatively convenient but may poorly represent a nasal compartment; cytology, lavage, absorptive matrices, and brushing differ in source resolution, dilution, and processing; biopsy retains architecture but is more invasive. Deployment requires tolerable sampling burden, acceptable turnaround time and cost, and standardized preanalytics. Analytical validity includes precision, stability, limits of detection, and interlaboratory agreement. Classifiers require prespecified thresholds, discrimination and calibration, donor-independent external validation, and incremental decision value over history, sensitization, symptoms, airflow, and cytology where available. Calibration applies to predicted probabilities, while assay calibration addresses measurement standardization. Age- and exposure-specific transportability must also be tested.

A cross-sectional study of 118 adults compared HDM-specific IgE harvested by nasal brushing with results from nasal challenge. The reported diagnostic area under the curve was 0.93; among systemically sensitized participants, a study-derived cutoff above 0.14 kUA/L had 92% sensitivity and 86% specificity (69). This is a diagnostic example within a defined population. The cutoff should not be transferred to systemically negative patients with local AR, children, other allergens, or treatment selection without independent validation.

### 7.4 Avoiding circular endotype discovery

An IL-13-high cluster validated by other IL-13-responsive genes is internally coherent, but this does not establish independent causality; a classifier separating obvious AR from healthy controls likewise does not establish an endotype classifier. Multiomic integration can generate hypotheses (70), but discovery, mechanism testing, and clinical validation require separate data and endpoints. The participant is the unit of replication, and external calibration against routine information matters more than high internal cross-validation.

### 7.5 Comparison with established AR classifications

Existing approaches answer complementary questions. Systemic, local, and dual sensitization classifications describe where allergen reactivity is demonstrable (12, 68). Eosinophil-, neutrophil-, or cytokine-associated profiles describe sampled inflammatory patterns (57). Pediatric onset trajectories describe longitudinal clinical and blood-transcriptomic variation (13). State–Place–Time adds explicit sampling location, response phase, and the evidence needed to move from observation to dependence or utility. It does not replace these schemes, provide validated cutoffs, or prove that the proposed examples are distinct patient groups. A cross-sectional inflammatory cluster remains informative, but its meaning may change with recent exposure, medication, comorbidity, or sampling modality.

A prospective chronic-rhinitis study of 259 patients and 20 controls identified six clusters combining clinical features with local eosinophils and local/serum IgE, including conventional AR, eosinophilic nonallergic rhinitis, local AR, and idiopathic rhinitis (71). These clinically useful distinctions organize observed inflammation; the study did not selectively test each cluster’s mechanistic dependence. A 10-year follow-up of 176 local-AR patients and 115 controls supported a persistent local-allergic phenotype without excess conservation to systemic atopy (72). Longitudinal phenotype persistence is distinct from proof that the same cellular dependency or tissue-resident clone persists. State–Place–Time can annotate these established categories with specimen, exposure phase, and the missing validation step rather than relabeling them.

## 8 Study designs that can close the evidential chain

### 8.1 Discovery must be exposure-resolved and donor-replicated

Discovery cohorts need enough clinical resolution to distinguish predisposition from current exposure. Allergen, season, recent symptoms, medication, asthma, sampling site, and disease control belong in the design rather than being addressed through *post hoc* explanation. Healthy controls, symptomatic nonallergic rhinitis, local allergic rhinitis, and asymptomatic sensitized individuals test different contrasts and cannot be pooled as a generic comparator. Additional modalities are justified when they close a specific gap—for example, surface protein for receptor engagement or receptor sequencing for local clonal recall—not simply because they increase dimensionality.

The participant, not the cell or spatial spot, remains the unit of biological replication. A candidate state should be defined in one donor set, reproduced in another, and then localized with a targeted method. Further subdivision of clusters in the discovery cohort cannot substitute for those steps.

### 8.2 Serial challenge can connect state, place, and time

Controlled nasal allergen challenge converts a case–control contrast into a within-person perturbation. Sampling at baseline, within minutes, at 2–8 h, and during recovery distinguishes response potential, immediate activation, late recruitment, and resolution. The clinical output—itch, secretion, exudation, or airflow—must be measured at the same time as the molecular state. Repeated blood, brushing, and lining-fluid sampling can define the trajectory; biopsy or spatial imaging should be restricted to the phase in which the proposed anatomical relation is expected.

Repeated standardized challenges or a prospective natural-exposure period should test whether a response recurs within a patient. Models should separate technical repeats, within-person variation, and between-person differences. Stability need not mean continuous marker elevation: a reproducible inducible trajectory can identify a consistent dependency. Independent repeat classification, not repeated measurements within one exposure episode alone, is required before proposing a stable patient-level label.

In a small Timothy grass study of 15 allergic participants and 12 controls, exposure-chamber responses at 120 min correlated more strongly with peak-season symptom/medication scores than did responses to the short nasal challenge (73). Challenge route, dose, duration, and endpoint therefore require explicit definition. These exposure-dependent differences do not constitute external validation of a state–place–time classifier.

### 8.3 Matching human perturbation to the claim

Perturbation has to match both the claim and the response phase. Patient-matched coculture can test whether a nominated fibroblast signal alters a prespecified T-cell outcome; a basal-cell memory claim requires persistence after cytokine withdrawal and an altered differentiation or rechallenge response. These experiments establish direction and capacity within the reconstructed system. They cannot demonstrate effects that require innervation, blood flow, recruitment, or glandular physiology.

Intact-system dependence is stronger when a selective intervention changes the nominated state and its linked outcome at the predicted time while leaving unrelated phases comparatively intact. Broad corticosteroid responses are biologically important but provide weak evidence for attributing causality to one of several simultaneously suppressed programs.

Priorities include donor-matched epithelial–immune or fibroblast–T-cell systems with withdrawal and rechallenge; paired blood–nasal TCR and B-cell receptor (BCR) tracking to separate local persistence from recruitment; and selective pathway blockade with matched spatial and timed physiological outputs. Candidate treatment-selection markers should then be evaluated in prospective randomized comparisons with prespecified interaction analyses, rather than being inferred from posttreatment molecular change.

### 8.4 Keeping computation within the evidence

Ligand–receptor databases, pseudotime, regulatory-network inference and machine learning can prioritize the next test, but they do not repair a missing link in the evidence chain. Models must avoid cell-level pseudoreplication and data leakage across donors. Predictive classifiers require external validation, calibration, and comparison with routine clinical variables. Separation of an existing case–control cohort is insufficient; the model has to improve a decision for a new patient.

Pseudotime estimates a plausible ordering of states, not directly observed lineage progression. Chromatin accessibility marks regulatory potential, not necessarily transcription-factor activity. Ligand–receptor compatibility identifies a possible interaction, not actual binding or interaction direction, and spatial deconvolution estimates composition based on reference assumptions. Their value lies in narrowing alternatives and specifying a falsifiable experiment, not in restating an association as a mechanism.

## 9 Clinical translation: begin with the unresolved decision

These scenarios illustrate research questions when routine assessment leaves a clinically important uncertainty. They are not validated diagnostic or escalation algorithms. The framework is research-oriented and complements established ARIA–EAACI guideline-based care; it does not replace it (1–3). Assessment and treatment remain based on clinical history, relevant sensitization, disease burden and control, appropriate intranasal therapy, and AIT in selected patients. Research measurements should be evaluated against these routine comparators at a predefined decision point.

### 9.1 Four decisions, not four new phenotypes

#### 9.1.1 Diagnostic discordance

When systemic tests are negative despite an allergen-specific history, the question is whether a defined allergen drives the nasal response. Nasal challenge supplies controlled exposure and a timed outcome. Local IgE or basophil activation can provide support, but negative results cannot reliably exclude local AR (12, 68). A proposed assay should be tested in this uncertain population for diagnostic accuracy and incremental decision-making value, rather than evaluated by its ability to distinguish obvious AR from healthy controls. Nasal brushing IgE offers a concrete diagnostic candidate (69), but its internally derived adult, systemically sensitized cutoff does not resolve the distinct scenario of systemic test negativity.

#### 9.1.2 Delayed obstruction after immediate control

Standard management and alternative causes of obstruction remain the first assessment. Research challenges can relate early and late airflow measurements to cytokines and cytology (33, 34). A candidate late-phase marker needs repeat assessment of its classification and a validated threshold; a randomized marker-by-treatment comparison would be needed before using it to select escalation. The current IL-13 observations do not justify biomarker-directed treatment.

#### 9.1.3 Disproportionate watery rhinorrhea

Glandular secretion, plasma exudation, inflammation, and nonallergic triggers may contribute in different ways. Source-related secretion measurements and guideline-consistent clinical assessment are the comparators for a proposed neural marker. Improvement after a clinician-directed anticholinergic treatment is compatible with a glandular contribution; it neither proves a generalized neuroimmune endotype nor validates a molecular classifier.

#### 9.1.4 Persistent symptoms during AIT

Allergen coverage, adherence, dosing, exposure, structural obstruction, and nonallergic triggers must first be assessed. Paired nasal measurements can then assess target engagement in research. A global molecular shift constitutes pharmacodynamic evidence unless a pretreatment or early marker prospectively predicts outcome and adds decision value. Selection between treatment options additionally requires evidence of differential benefit (41, 63, 64). In a 1-year cockroach SCIT trial in children with asthma and nasal challenge reactivity, the SCIT and placebo groups analyzed (28 and 29 participants) had no differential improvement in nasal symptoms despite a large increase in serum IgG4 and other systemic effects (74). This is a population- and regimen-specific counterexample to using systemic target engagement as a surrogate for nasal benefit; it does not establish general AIT failure.

### 9.2 Therapeutic implications follow the maturity of evidence

Therapeutic implications should be proportional to evidence obtained directly in AR. Anti-IgE trials support IgE-pathway dependence in defined populations (29, 30), but a molecular tissue framework does not by itself establish an indication or select a biologic. Findings from asthma or CRSwNP should not be transferred to routine AR management without relevant evidence. Barrier-directed and stromal strategies remain research hypotheses; matched perturbation is proposed as a validation step, not a current treatment recommendation.

Modules can overlap, so the practical target is the residual dependency after standard care. If an antihistamine removes itch but not delayed obstruction, the residual symptom becomes a cleaner target for anti-inflammatory testing. This sequential logic is more informative than assuming that one pathway explains the whole disease.

### 9.3 A proportionate distinction between clinical tools and research measurements

Most patients do not require molecular tissue profiling. History, sensitization, symptom timing, objective airflow, nasal cytology where available, and response to targeted treatment will usually define the relevant uncertainty. Nasal allergen challenge and local specific IgE may add information in selected diagnostic settings. Serial cytokine measurements, epithelial panels, spatial profiling, and multiomic assays should remain research tools until they improve a defined clinical decision beyond these simpler measures.

## 10 Discussion

State–Place–Time organizes nasal observations by cellular program, anatomical setting, and response phase. Its contribution is to identify the missing test needed to distinguish a tissue description from a mechanistic or clinical claim. IgE-pathway dependence, timed late responses, epithelial feedback, and separable physiological outputs illustrate different evidence links; stromal maintenance remains a low-maturity boundary case.

The framework itself must be testable. In independent donor-level studies, a prespecified model combining state, place, and time should be compared with simpler state-only and routine clinical models for the same outcome. Consistently absent incremental value in explaining, predicting, or informing decisions after appropriate measurement would weaken the framework’s proposed practical advantage. Reproducible evidence that localization or phase does not modify the nominated relationships would challenge those organizing assumptions. A negative individual pathway experiment does not refute every tissue dimension, and a failed classifier may reflect measurement error; these alternatives must be evaluated without making the model immune to negative results.

The priorities are repeated within-patient challenge with paired molecular, spatial, and physiological sampling; donor-matched withdrawal/rechallenge and selective perturbation; blood–nasal clonal tracking; and external biomarker validation followed by prospective treatment-interaction testing. Discovery, mechanism testing, and clinical validation should use distinct endpoints and independent donors where possible.

Limitations include purposive narrative selection, incompletely recoverable initial search and screening records, subjectively selected domains and conceptual appraisal, and source availability. Retrieval from three databases improves breadth but does not establish exhaustive coverage: search vocabulary and the 2016–2026 update window can miss relevant work, and Web of Science edition coverage depends on institutional access. No complete eligibility screening, formal risk-of-bias assessment, or validated evidence grade is claimed. Small cohorts, heterogeneous platforms and comparators, context-limited surgical tissue, and experimental extrapolation constrain inference. Registry records are not primary mechanistic results. These limitations favor cautious propositions and explicit next tests; the five examples do not exhaust AR biology.

## 11 Conclusions

Nasal tissue heterogeneity can be examined through cellular programs, anatomical setting, and response phase. The proposed framework distinguishes these descriptions from demonstrated dependence and validated patient-level utility. IgE–FcϵRI has strong pathway evidence, while late-phase and epithelial findings require further longitudinal and clinical validation. Sensory, vascular, and secretory outputs are separable; stromal maintenance remains hypothetical. Clinical implementation requires reproducible markers and additional decision value, with evidence of differential benefit for treatment selection.
