# Supplementary material for: Toward mechanistic endotyping in allergic rhinitis: a State–Place–Time framework for appraising nasal tissue evidence
Source: Front Immunol. 2026 Oct 13;17:1972016. doi: 10.3389/fimmu.2026.1972016 (PMC13711116; doi:10.3389/fimmu.2026.1972016)
Supplement: Supplementary file 1 [file Table1.docx]

Supplementary Table S1

Representative evidence informing the State–Place–Time framework. Each row identifies disease context and distinguishes human observation, human-derived function, indirect/model evidence, and inference. The table is purposive, not an exhaustive systematic-review inventory. Assay-specific denominators are stated where verified from accessible full text or supplementary material. “Not reported” means that the checked accessible primary record did not state the requested item; inaccessible supplementary files are identified explicitly, and no denominator is inferred from them.

| **Study and reference** | **Population comparator and sample size** | **Study design sampling method and site** | **Exposure phase and major finding** | **Evidence category and key limitation** |
| --- | --- | --- | --- | --- |
| Ruysseveldt 2024 Reference 6 10.1016/j.jaci.2024.04.021 | Inferior-turbinate donors. scRNA-seq: control 2/HDM-AR 2; total-basal-cell flow: 19/7; KRT5 imaging: 5/5; HRH1: 7/7; PAR-2: 6/4; ITGA2hi flow: 5/7; AR-cell proliferation: n=5; ALI/TEER: 7/7; scratch area: 12/12 (closure-rate subset 6/6). | Cross-sectional nasal scRNA-seq; flow cytometry, imaging and primary-cell proliferation/wound/air–liquid-interface assays. | Clinical tissue state; culture perturbation. Altered basal-cell subtypes and progenitor/barrier capacity. | Direct AR association + human-derived function. Denominators vary by assay and must not be replaced by a single overall n; cross-sectional direction remains unresolved. |
| Zhao 2026 Reference 8 10.3389/fimmu.2026.1788288 | Nasal polyposis with/without AR. Methods: 9/group; abstract: 10/group; unresolved. | Surgical nasal-polyp-associated mucosa; targeted Xenium, qPCR, adjacency and communication inference. | Surgical cross-sectional state; recent steroid exclusion described. Fibroblast-associated organization. | Context-limited human spatial association + inference. Polyposis and reporting discrepancy; no selective maintenance test. |
| Liu 2026 Reference 7 10.1016/j.jaci.2026.06.020 | 49 participants in total. scRNA-seq: 25 AR/14 symptomatic nonallergic rhinitis (39); scATAC-seq subset: 16/8 (24); additional immunofluorescence cohort: 5/5 (10). AR required an ARIA diagnosis, positive skin-prick test (>3 mm) or allergen-specific IgE >0.35 U/L, and disease duration >3 years. Systemic corticosteroids, immunosuppressants or biologics within 4 weeks were exclusionary; sampling season, current allergen-exposure phase, and intranasal-corticosteroid/antihistamine washout were not reported. | Endoscopically obtained nasal mucosal tissue; exact anatomical subsite not reported. scRNA-seq/scATAC-seq, immunofluorescence, communication, trajectory, regulatory models and classifier. | Cross-sectional tissue state; sampling season and allergen-exposure phase were not reported. Epithelial maturation and stromal programs differ. | Direct AR/NAR association + inference. The comparator had symptomatic NAR rather than healthy mucosa. Missing exposure context, intranasal-steroid/antihistamine washout and exact sampling subsite limit baseline comparability; status prediction is not causal or treatment-selection validation. |
| Kim 2026 Reference 14 10.1016/j.jaci.2026.02.019 | 48 patients undergoing surgery for chronic rhinosinusitis, sinonasal tumor or septal deviation. Same-site paired brushing/tissue: scRNA-seq 4 pairs; flow cytometry 34 pairs; non-polyp subset 10 pairs; fresh-versus-frozen subset 9 pairs. | Paired brushing and tissue obtained endoscopically from the same nasal site; exact subsite was not specified. Recent systemic/intranasal corticosteroid or antibiotic use (within 1 month) was excluded. | Paired sampling-method comparison. Surface versus deeper cell recovery differs. | Indirect, heterogeneous sinonasal surgical-cohort evidence—not an AR-only cohort. Quantitative cell proportions should not be transferred to uncomplicated AR. |
| Steelant 2018 Reference 16 10.1016/j.jaci.2017.08.039 | Human groups: healthy controls 10, HDM-AR 9 and idiopathic rhinitis 6. Tissue barrier analyses use these clinical groups; nasal-fluid/Calu-3 barrier experiment n=5/group and anti-IL-4Rα experiment n=5/group. | Nasal secretions; human nasal tissue resistance/permeability and junction assays; primary epithelial/Calu-3 mediator and blockade experiments; complementary mouse tests. | Short-term culture exposure and model inflammation. Mediators impair epithelial barrier properties. | Human association + human-derived function + animal mechanism. Cell-supernatant results include repeated experiments and should not be interpreted as independent clinical donor numbers; mouse blockade does not establish human selective dependence. |
| Gao 2026 Reference 24 10.1016/j.waojou.2026.101336 | Swabs: 17 HDM-AR/17 controls; secretions: 15/15. Biopsy recruitment: 8 CRSwNP+AR/22 CRSwNP without AR/8 controls. | Inferior-turbinate swabs; middle-meatus absorptive secretions; surgical biopsies. ALI culture and nasal-cell stimulation partly CRSwNP-derived. | Cross-sectional clinical sampling; HDM/PAR-2 perturbation in culture. SCC/IL-25-related epithelial signals. | Direct AR swab/fluid association + context-limited polyp tissue/culture. Tissue-staining subset differs from recruitment; no obligatory intact-AR circuit. |
| Corren 2023 Reference 28 10.1016/j.jaci.2022.08.029 | 121 cat-allergic patients randomized among combination, SCIT, tezepelumab and placebo. | Double-blind parallel trial; nasal challenge, skin tests, blood/nasal samples and epithelial transcriptomics. | 52-week treatment + 52-week observation. Combination improves on-treatment challenge response. | Direct human intervention. Primary year-after-withdrawal symptom AUC not superior to SCIT; pathway engagement does not define a predictive endotype. |
| Lin 2004 Reference 29 10.1016/j.jaci.2003.11.044 | 24 ragweed-allergic rhinitic participants; omalizumab versus placebo. | Randomized double-blind 6-week challenge trial; nasal volume, serum IgE and blood-basophil FcεRI. | Baseline and repeat challenge during treatment; receptor measures days 7–42. Nasal response attenuation. | Direct human pathway intervention. Blood FcεRI is not a source-resolved nasal tissue marker; no marker-guided treatment-selection comparison. |
| Okubo 2020 Reference 30 10.1016/j.jaip.2020.04.068 | Severe cedar pollinosis: omalizumab 162/placebo 175, with standard care. | Randomized double-blind phase III add-on trial; nasal/ocular symptoms and quality of life. | Seasonal disease during standard care. Add-on clinical benefit in this defined population. | Direct human AR intervention. Population-specific efficacy; no tissue-proximal marker or validated treatment interaction. |
| Leaker 2017 Reference 33 10.1038/mi.2016.74 | 19 hay-fever participants; 12 show late cytokine elevations. Comparator/intervention subsets not completely specified in abstract. | Grass-pollen nasal spray challenge; nasosorption, nasal curettage, proteins and transcriptomics; prednisone perturbation. | Serial immediate/late sampling. Parallel type 2, IL-1 and complement-associated changes. | Direct human timing + broad intervention. Prednisone is not pathway-selective; independent recurrence/clinical utility unresolved. |
| Campion 2023 Reference 34 10.1016/j.jaci.2023.06.026 | Birch-pollen allergy: challenge 20/placebo 10; 8/20 IL-13-high responders. | Randomized double-blind challenge on 3 consecutive days; nasal secretions, 33 mediators, symptoms and airflow. | Days 1 and 3: selected points over 24 h. IL-13 rise at 2–8 h with later secondary airflow reduction. | Direct human temporal association. Independent test–retest classification and externally validated threshold/interaction not established. |
| Xie 2021 Reference 35 10.1111/all.14835 | 10 participants with AR AND mild asthma; sequential intranasal steroid/placebo phases. | Single-blind placebo-controlled sequential study; 14-day pretreatment, 7-day washout; mucosal/blood flow cytometry and lavage. | 24 h after nasal challenge. ILC2/type 2 responses attenuated by steroid. | Comorbid AR human intervention. Broad steroid effect, small n; no ILC2-selective causal test. Design is sequential, not described as randomized crossover. |
| Thornton 2013 Reference 36 10.1016/j.clim.2013.02.008 | Persistent AR 12/seasonal AR 7/controls 10. | Inferior-turbinate biopsies before and 8/48 h after allergen; bradykinin reflex secretion testing. | Eosinophil–nerve localization at 8–48 h; increased reflex secretion at 24 h. | Direct human spatial/temporal association. No selective eosinophil–nerve blockade; source direction not established. |
| Skrindo 2015 Reference 40 10.1111/cea.12543 | Study totals: grass-pollen AR 13, birch-pollen AR 11 and nonallergic controls 16. Grass-extract explants: 13 AR/8 controls; birch-extract and Bet v 1-peptide explants: 11 AR/8 controls. Flow cytometry: 6 allergic/5 controls; grass-explant tryptase subset: 5/4; basophil activation control experiment: 2/2. | Lower-turbinate biopsy explants obtained outside pollen season; relevant extract or Bet v 1 peptide mix; 24-h culture mediator measurement. | Off-season ex-vivo stimulation. Local allergen recall and IL-5 production. | Human-derived function with assay-specific denominators. Excludes new recruitment during culture but does not prove canonical tissue residence or intact-nose necessity; approximately half of allergic explants produced detectable IL-5, emphasizing response heterogeneity. |
| Testera-Montes Reference 11 10.1111/all.15292 | Local AR 9/conventional AR 5/nonatopic controls 5. | 3-day HDM nasal challenge; paired biopsy/blood, immunohistochemistry, recombination markers and B-cell assays. | Before/after provocation. Plasmablast changes compatible with local sequential IgE switching. | Direct human association + cellular stimulation. Small cohorts; local synthesis/recruitment and durable memory require further resolution. |
| Raphael 1991 Reference 58 10.1016/0091-6749(91)90298-3 | 9 atopic seasonal-rhinitis participants; within-person side comparison. | Unilateral allergen challenge; bilateral lavage for protein and mediators. | 7-hour serial sampling; protein differences through early hours. Ipsilateral exudative versus contralateral gland-related pattern. | Direct human physiological association. Compatible with reflex/gland and vascular separation; not a complete cell-resolved circuit. |
| Orban 2021 Reference 62 10.1111/cea.13775 | 12 moderate–severe AR/16 healthy controls; within-AR diluent then allergen. | Five alternate-day diluent challenges, four-week interval, five allergen challenges; secretions and biopsies. | Biopsy 24 h after first/fifth challenges. Priming and inflammation without measured structural remodeling. | Direct human repeated-exposure evidence. Limited duration and tested structures; no proof that remodeling never occurs. |
| Altman 2023 Reference 41 10.1016/j.jaci.2023.06.025 | Molecular cohort in GRASS: placebo 30/SCIT 27/SLIT 27 (84 total); assay-specific availability varies. | Longitudinal nasal-brush and PBMC transcriptomics after challenge in randomized parent trial. | Annual specimens during 2 treatment years and 1 follow-up year. Molecular changes associated with symptoms/airflow. | Direct human pharmacodynamic association. These 84 participants are not the 106 randomized parent-trial denominator; no pretreatment predictive interaction. |
| Scadding 2017 Reference 65 10.1001/jama.2016.21040 | 106 randomized: SLIT 36/SCIT 36/placebo 34; 92 completed year 3. | GRASS randomized double-blind trial; challenge and symptoms at baseline and years 1–3. | Two treatment years then one year off treatment. SLIT vs placebo primary year-3 response not different. | Direct human intervention/withdrawal. Two-year regimen result; not a general failure of guideline-duration AIT. SCIT comparison not powered. |
| Shin 2024 Reference 13 10.1016/j.jaci.2023.12.024 | Birth cohort 1,050: early onset 88, intermediate transient 110, late onset 209, very late onset 187 and never/infrequent 456. Blood-transcriptome subset n=36: early onset 12, late onset 12 and never/infrequent 12; mean age at blood collection 6.58, 6.75 and 6.92 years, respectively. | Clinical trajectory analysis from birth to 12 years; PAXgene peripheral-blood transcriptomes using GeneChip Human Gene 2.0 ST arrays. | Developmental/onset timing. Distinct clinical trajectories and systemic transcriptomic associations. | Human age-modifier evidence, indirect for nasal tissue. The 1,050 trajectory cohort and 36-child transcriptome subset are reported separately; blood programs do not validate pediatric nasal endotypes or adult thresholds. |
| Ma 2026 Reference 57 10.3389/fimmu.2026.1831730 | AR 40/NAR 40/controls 20; adults, retrospective records. | Nasal secretions and brush cytology; local inflammatory profiles, correlations. | Cross-sectional clinical records, 2023–2025. Type 2 versus neutrophil-associated profiles. | Human descriptive classification. No nasal challenge to exclude local AR in NAR; methods/results comorbidity reporting is not fully consistent. |
| Zhang 2026 Reference 67 10.4193/Rhin25.381 | 60 HDM-AR/143 controls recruited February 2023–September 2024 under the same protocol. Washout/exclusion: systemic corticosteroids 3 months, intranasal corticosteroids 4 weeks, antihistamines 2 weeks, topical decongestants 1 week and monoclonal antibodies 6 months. | Bilateral cotton-piece sampling from the middle nasal meatus for 5 min; local/serum Der p and Der f IgE; nasal cytokines and diagnostic models. | Sampling spanned seasons; exact exposure phase and case–control season matching were not reported. Local IgE associated with type 2 signals. | Direct human association. Defined medication windows improve interpretability, but unreported exposure/season matching limits baseline comparison; case/control performance does not establish treatment selection. |
| Hoang 2026 Reference 68 10.4193/Rhin25.237 | 21 included diagnostic studies; participant counts vary by underlying study. | Meta-analysis/indirect diagnostic comparison: local nasal IgE, basophil activation and nasal eosinophilia against provocation. | Diagnostic assessment; no uniform phase across component studies. Limited sensitivity supports challenge follow-up when negative. | Secondary diagnostic evidence, not independent mechanistic primary replication. Heterogeneity and indirect test comparisons; no treatment utility. |
| Wils 2024 Reference 25 10.3389/fimmu.2024.1394539 | AR/CRSwNP/controls; scRNA-seq 2 AR+2 controls; flow cytometry 5/group; assay subsets vary. | Inferior-turbinate or polyp-derived tissue; molecular/imaging assays, RNA scope, flow and scRNA-seq; cold-dry-air physiology. | Clinical tissue and provocation. Little evidence of a substantial nasal neuroendocrine-cell population. | Direct/context-limited human negative observations. Small assay subsets and rare-cell detection limits do not establish universal absence. |
| Qi 2025 Reference 47 10.4168/aair.2025.17.4.486 | AR versus controls; clinical sampling counts require assay-level full-text verification. | Clinical mucosal NMU/NMUR1/ILC2 expression, nasal-cell stimulation, and OVA mouse intervention. | Clinical association plus ex-vivo and experimental exposure. NMU-associated ILC2/type 2 responses. | Human association + human-derived function + animal mechanism. Mouse NMU/ERK perturbation cannot prove intact human circuit or clinical benefit. |
| Jafari 2025 Reference 51 10.1002/clt2.70097 | 23 birch/timothy AR patients; paired before/after SCIT updosing. | Blood, nasal lavage and inguinal-node fine-needle aspirates; antibody and B-cell assays. | Early treatment trajectory. Local/systemic immunoglobulin changes diverge. | Direct human longitudinal association. No untreated randomized comparator; early changes do not establish durable tolerance or predictive treatment benefit. |
| Deng 2026 Reference 22 10.1016/j.bjorl.2026.101800 | 30 HDM-AR/30 controls; protein assays n=12 and RNA assays n=30 as reported; culture donor replication differs. | Surgical turbinate/septal-context nasal tissue; junction-associated proteins and S100A8; HDM/siRNA in human epithelial cells. | Clinical cross-sectional state plus timed culture exposure. S100A8 association and protein restoration after silencing. | Direct human association + human-derived perturbation. Junction-associated protein recovery alone does not establish functional barrier or clinical efficacy. |
| Ruysseveldt 2026 Reference 23 10.1016/j.mucimm.2026.02.004 | HDM-AR with active disease and skin-prick positivity versus nonallergic controls. Inferior turbinates were partly removed during functional/aesthetic rhinoplasty. ALI/TEER: control 7/AR 7; other assay denominators are not stated in the accessible supplementary captions. | Inferior-turbinate biopsies and primary basal cells; Der p 1/PAR-2, mast-cell mediator, proliferation, wound-healing, differentiation and barrier assays. | Cross-sectional tissue plus cell stimulation. Inflammatory basal-cell responses and impaired progenitor functions. | Direct association + human-derived function. Assay-specific denominators should be read from each figure/source-data file; TEER n must not be generalized to all assays. Reciprocal capacity does not prove a sustained in-vivo loop. |
| Meng 2019 Reference 71 10.1111/all.13640 | 259 chronic-rhinitis patients/20 controls; mixed clinical categories. | Prospective clinical and nasal inflammatory assessment; 12 clinical variables, 5 inflammatory measures, cluster analysis. | Cross-sectional clustering; local eosinophils and local/serum IgE distinguish six groups. | Human descriptive classification. No cluster-specific selective perturbation or treatment-selection validation. |
| Hamizan 2025 Reference 69 10.12932/AP-031122-1495 | 118 adults with rhinitis; systemically sensitized subgroup distinguished by NAC response. | Diagnostic cross-sectional study; nasal mucosal brushing HDM-specific IgE, systemic tests and NAC. | Diagnostic sampling; AUC 0.93; study-derived >0.14 kUA/L cutoff: sensitivity 92%, specificity 86% in sensitized subgroup. | Direct human diagnostic evidence. No independent cutoff validation or treatment interaction; not generalizable to systemically negative LAR. Final issue 2025, online 2023. |
| Liu 2021 Reference 52 10.1002/lio2.693 | 30 HDM-AR with septal deviation/21 septal-deviation controls; explant experiments n=6. | Surgical inferior-turbinate tissue; sequential-section IgE/CD138 staining, stress markers; 10 mmol/L 4-PBA explant culture. | Clinical state plus 12/24/48-hour perturbation; IgE reduced at 12/24 h, not 48 h. | Human association + human-derived function. Tissue inhibitor is not B-cell-specific; no durable memory, in-vivo dependency or clinical validation. Full-text/figure-7 counts checked. |
| Linton 2026 Reference 15 10.1016/j.jaci.2026.05.003 | 19 ragweed-AR/12 nonallergic controls; out-of-season challenge. | Middle-meatus/adjacent-cavity secretions, 16S sequencing; IgE and S. aureus carriage. | Baseline and 6/24/48 h; no overall diversity change with NAC; community differences associated with sex/carriage. | Direct human temporal/context evidence. Modest cohort; no causal host-factor test; genus-level changes differ from whole-community stability. |
| Zoratti 2024 Reference 74 10.1016/j.jaci.2024.04.022 | Children with asthma, cockroach sensitization and NAC reactivity; endpoint groups SCIT 28/placebo 29. | Randomized double-blind one-year SCIT/placebo; NAC TNSS, nasal transcriptome and systemic antibody/T-cell assays. | Treatment and repeat NAC; no differential nasal TNSS change (P=.63) despite systemic IgG4 increase. | Human intervention, asthma-comorbid pediatric context. Negative nasal outcome despite systemic engagement; not general AR or guideline-duration AIT failure. Endpoint denominator is not assumed to equal enrollment. |
| Rondón 2018 Reference 72 10.1111/all.13272 | 176 LAR patients/115 matched healthy controls. | Ten-year prospective follow-up; annual clinical/systemic tests; nasal challenge at baseline and years 5/10. | Longitudinal phenotype; systemic atopy conversion not higher than controls. | Direct human longitudinal phenotype evidence. Persistence of clinical LAR does not prove stable cellular mechanism or tissue-resident clonal memory. |
| Zemelka-Wiacek 2025 Reference 73 10.1111/all.16518 | 15 timothy-grass-allergic participants/12 controls. | Exposure chamber, nasal challenge and 14-day peak-season symptom/medication assessment; symptoms, nasal geometry/airflow. | Chamber 120-min responses vs NAC 15-min response correlations; stronger chamber–field correspondence. | Direct human exposure/endpoint evidence. Small cohort, differing protocols; not independent classifier or differential-treatment validation. Full-text methods checked. |
| Zhu 2018 Reference 61 10.1111/crj.12566 | 25 persistent-AR/16 healthy controls. | Open-label randomized-order histamine/LTD4 crossover nasal provocation; symptoms, resistance, lavage/sputum. | Two-week challenge interval; distinct resistance vs sneezing/discharge patterns. | Direct human mediator intervention. Exogenous response supports outputs but not endogenous source necessity or marker-guided therapy. |

Abbreviations: AR, allergic rhinitis; NAR, nonallergic rhinitis; HDM, house dust mite; CRSwNP, chronic rhinosinusitis with nasal polyps; ALI, air–liquid interface; AIT, allergen immunotherapy; SCIT, subcutaneous immunotherapy; SLIT, sublingual immunotherapy; PBMC, peripheral blood mononuclear cell; OVA, ovalbumin; NMU, neuromedin U; NMUR1, neuromedin U receptor 1; ILC2, group 2 innate lymphoid cell; ERK, extracellular signal-regulated kinase; SCC, solitary chemosensory cell; scRNA-seq, single-cell RNA sequencing; scATAC-seq, single-cell assay for transposase-accessible chromatin using sequencing; FcεRI, high-affinity IgE receptor; IgE, immunoglobulin E.
