# Supplementary material for: Toward mechanistic endotyping in allergic rhinitis: a State–Place–Time framework for appraising nasal tissue evidence
Source: Front Immunol. 2026 Oct 13;17:1972016. doi: 10.3389/fimmu.2026.1972016 (PMC13711116; doi:10.3389/fimmu.2026.1972016)
Supplement: Supplementary file 2 [file Table2.docx]

Supplementary Appraisal S2

Criteria and cell-level rationale for Figure 2. This is an author-derived qualitative appraisal, not a validated evidence-grading system, numerical score, or clinical classifier. Filled circles, triangles, and open circles summarize specified features and are not numerical ranks. Symbols should not be summed and do not support patient assignment. No pooled score, inter-rater reliability estimate, or formal certainty grade is claimed. Color is decorative; shape and wording carry the meaning.

# Dimension-specific criteria

## **Human evidence**

Filled: direct clinically characterized human AR evidence supports the specified observation or pathway. Triangle: small, comorbid, disease-context-limited, indirect, or incompletely replicated support. Open: the relevant human feature is not established in the reviewed evidence. Human association does not establish causality.

## **Spatial localization**

Filled: the relevant compartment or physiological target is measured in humans. Triangle: plausible positioning, partial imaging, or context-limited spatial evidence. Open: the proposed location is not established in the reviewed evidence. Localization of an output is distinct from a demonstrated source–recipient niche.

## **Timing/recurrence**

Filled: human timed exposure or intervention establishes the relevant phase or reproducible temporal pattern. Triangle: phase associations lack independently validated recurrence, persistence, or ordering. Open: timing, recurrence, or persistence is not established in the reviewed evidence. A timed response need not be a stable endotype.

## Matched perturbation

Filled: a human intervention tests the claimed pathway or output dependency. Triangle: broad suppression, human-derived capacity or indirect/model perturbation. Open: a selective relevant-system dependency test is unestablished. Intervention specificity and timing must match the claim.

## **Accessible measurement**

Filled: a feasible, reproducible measurement identifies the proposed dependency in the intended population. Triangle: a technically measurable candidate has unresolved analytical, source, threshold, or replication issues. Open: a validated, feasible measurement of the dependency is not established in the reviewed evidence.

## Clinical utility

Filled: externally demonstrated incremental decision utility for a defined use. Triangle: preliminary decision/outcome testing without completed validation. Open: tissue-marker utility beyond routine assessment is unestablished. Treatment selection specifically requires differential benefit; drug efficacy alone is insufficient.

# Handling disagreement and indirect evidence

Interpret differences by disease context, sampling modality, response phase, donor-level replication, and perturbation specificity. Do not upgrade uncomplicated-AR support from polyp or mouse findings. Where a proposition remains unresolved, retain partial or unestablished support and state the missing test. Negative detection is conditional on the assay and tissue sampled. A mechanism with treatment efficacy may still lack a useful predictive tissue marker.

# Rationale for every matrix cell

## IgE–FcεRI effector activity

| **Dimension** | **Symbol** | **Rationale and source** |
| --- | --- | --- |
| Human evidence | filled | AR anti-IgE/challenge evidence (29, 30); mechanism at pathway level. |
| Spatial localization | triangle | Immediate outputs have plausible tissue access; no complete source-resolved mast-cell target map (29, 36, 58). |
| Timing/recurrence | filled | Minute-scale allergic response and intervention kinetics in humans (29). |
| Matched perturbation | filled | Anti-IgE attenuates nasal responses in defined trials (29, 30). |
| Accessible measurement | triangle | Serum/local IgE and blood FcεRI are measurable, but incomplete nasal surrogates (11, 29, 67, 68). |
| Clinical utility | open | No validated tissue-proximal marker improves treatment selection beyond routine information. |

## Late-phase type 2 amplification

| **Dimension** | **Symbol** | **Rationale and source** |
| --- | --- | --- |
| Human evidence | filled | Human challenge links cytokine phase and airflow (33, 34). |
| Spatial localization | triangle | Fluid and curettage sample local responses without source-resolved obstruction anatomy (33, 34). |
| Timing/recurrence | triangle | Timed signal exists, but small cohorts and independent repeat classification remain unresolved (33, 34). |
| Matched perturbation | triangle | Prednisone/steroid suppression is broad; no validated IL-13-defined selective interaction (33, 35). |
| Accessible measurement | triangle | Nasal cytokines are measurable; threshold, repeat status and deployment unresolved (34). |
| Clinical utility | open | No externally validated incremental decision utility or treatment interaction for the tissue label. |

## Barrier–repair dysfunction

| **Dimension** | **Symbol** | **Rationale and source** |
| --- | --- | --- |
| Human evidence | filled | Convergent AR tissue and primary-cell observations (6, 7, 16, 22, 23). |
| Spatial localization | filled | Epithelial compartment and junction/basal features are directly sampled (6, 16, 22). |
| Timing/recurrence | triangle | Cross-sectional and cell timing do not resolve intact-human antecedence or sustained feedback. |
| Matched perturbation | triangle | Human-derived restoration/cytokine/PAR-2 tests show capacity; no selective intact-AR clinical dependence (16, 22, 23). |
| Accessible measurement | triangle | Brushing/protein panels are candidates; analytical, repeat and clinical thresholds unvalidated. |
| Clinical utility | open | No validated panel demonstrates incremental clinical utility. |

## Sensory vascular and secretory output family

| **Dimension** | **Symbol** | **Rationale and source** |
| --- | --- | --- |
| Human evidence | filled | Human challenge physiology and biopsies support separate outputs (36, 58). |
| Spatial localization | filled | Gland-related secretion/exudation and nerve-associated observations distinguish compartments (36, 58). |
| Timing/recurrence | filled | Unilateral timed secretion and postchallenge nerve observations define phases (36, 58). |
| Matched perturbation | triangle | Human output-selective pharmacology is plausible, but this bundled row is not one selectively tested cellular mechanism. |
| Accessible measurement | triangle | Physiology can measure outputs; no validated combined molecular endotype marker. |
| Clinical utility | open | Existing symptom-directed care does not validate a new tissue-marker family or classifier. |

## Putative stromal maintenance

| **Dimension** | **Symbol** | **Rationale and source** |
| --- | --- | --- |
| Human evidence | triangle | AR/NAR association and polyp-context spatial evidence (7, 8); no maintenance dependence. |
| Spatial localization | triangle | Spatial pattern measured in nasal-polyposis tissue; limited transfer to uncomplicated AR (8). |
| Timing/recurrence | open | No demonstrated off-exposure stromal persistence in uncomplicated AR. |
| Matched perturbation | open | No direction-specific intact-AR stromal perturbation. |
| Accessible measurement | open | No validated accessible patient-level stromal-maintenance marker. |
| Clinical utility | open | No validated stromal label predicts or improves a clinical decision. |

# Revision-stage evidence reassessment

New diagnostic nasal-brushing IgE evidence (69) supports measurement and diagnostic promise but does not validate tissue treatment selection. Local IgE explant inhibition (52) supports functional capacity, not enduring memory. Pediatric SCIT systemic–nasal discordance (74) reinforces the missing clinical-surrogate link. Mediator challenge (61) supports separable outputs without identifying one endogenous source circuit. The clinical-utility cells remain unestablished; no symbolic grade is upgraded solely by adding citations.
