# Supplementary material for: Toward mechanistic endotyping in allergic rhinitis: a State–Place–Time framework for appraising nasal tissue evidence
Source: Front Immunol. 2026 Oct 13;17:1972016. doi: 10.3389/fimmu.2026.1972016 (PMC13711116; doi:10.3389/fimmu.2026.1972016)
Supplement: Supplementary file 3 [file Table3.docx]

Supplementary Methods S3

Revision-stage retrieval and focused narrative evidence appraisal. This supplement distinguishes recoverable initial web-search history from executed database updates. Counts describe records, not systematically included studies.

# Initial work and date correction

The initial search documentation did not retain a reproducible native PubMed strategy, database hit count, or complete selection flow. The unsupported August 1 cutoff was therefore removed rather than retrospectively certified. Available original documentation is retained by the authors and can be provided on request. The revision-stage database searches reported below were conducted prospectively to improve transparency; they do not convert the article into a systematic review.

# Databases, dates, fields and restrictions

PubMed/MEDLINE: NCBI E-utilities, executed September 18, 2026; publication dates January 1, 2016–September 18, 2026. Five overlapping queries yielded 1,099 instances and 823 unique PMIDs. Embase: Elsevier interface accessed institutionally, executed September 18, 2026; publication years 2016–2026; explicit Emtree explosion plus title/abstract/keyword fields. Total 1,134 comprised 1,093 bibliographic records and 41 trial-registry records. Web of Science: Core Collection, interface Editions: All, executed September 18, 2026; 977 unique UT identifiers, publication years 2016–2026; Topic fields include title, abstract, author keywords and Keywords Plus. The institutional indexes available under All are not enumerated in the exports. No additional language, document-type, or species restrictions were applied. Retrieval included reviews and conference/preprint records; retrieval itself does not establish primary-study eligibility. Scopus was not searched because Embase and Web of Science were selected as the two additional multidisciplinary/biomedical sources requested by the reviewer.

# Record reconciliation and deduplication

The bibliographic sets total 2,893 instances (823+1,093+977). DOI or PMID matches and exact normalized titles were used to consolidate reports; conflicting nonempty DOIs prevented title-only matching. This removed 1,617 duplicate instances and produced 1,276 unique bibliographic records. It is report-level matching, not consolidation of every publication from one clinical study. Metadata variants may remain. The 41 registry records were kept separate and were not counted as published trial results. The earlier 1,075-record Embase export is a subset of the final 1,093-record export and was not added again. Incorrect-topic or blank downloads were excluded from the source set.

# Selection, exclusions and appraisal actually performed

All merged records underwent metadata-assisted prioritization. Titles and available abstracts were searched for terms representing the reviewer-identified gaps: existing AR endotype/classification systems; single-cell or spatial evidence; epithelial barrier and repair; local IgE/B-cell memory; ILC2 and dendritic-cell inputs; neuroimmune, vascular, and secretory outputs; exposure timing; and biomarker validation. Matches to the original reference list, disease-context signals, and publication-status flags were also recorded. This procedure ranked records for narrative review; it was not a complete eligibility screen. From records outside the executed PubMed query set, 25 candidates with the clearest direct relevance to those gaps underwent focused title/abstract appraisal. Eight published papers were added as references 15, 52, 61, 69, and 71–74. The other 17 were not added because the disease context was indirect, the content duplicated retained evidence, the report was secondary diagnostic synthesis, or it did not address the nominated mechanistic or validation gap. The audit log records every decision and its evidence boundary. These are purposive narrative addition decisions, not systematic-review exclusions. Earlier landmark studies outside the recent-window retrievals were retained through reference checking as historical or conceptual context; they were not counted as revision-stage search hits. Existing citations and earlier focused checks were retained; overlapping activities must not be summed as independent screened-study counts.

Considered primary sources addressed characterized human nasal tissue/physiology, controlled exposure, longitudinal trajectories or relevant human-derived functional tests. Priority went to a specific reviewer gap and an experiment interpretable by population, site, phase and endpoint. Related-disease and animal findings were explicitly indirect; systemic biomarkers alone were not nasal dependencies. Unrelated reports, nonspecific treatment outcomes without relevant mechanistic information, unpublished observations and registry summaries were not primary mechanistic anchors. Reviews/guidelines informed context and reference tracing. Language appraisal depended on accessible records/texts; no retrieval language limit does not imply every language was assessed. Conference/preprint, withdrawal/concern and unavailable-abstract records were flagged (157 records), not automatically excluded by a purported formal screen. No dual independent screening, registered protocol, formal risk-of-bias score, PRISMA flow, or complete full-text assessment of all 1,276 records is claimed.

# Exact executed PubMed strategies

## Cellular and spatial — 24 records

("Rhinitis, Allergic"[Mesh] OR "allergic rhinitis"[Title/Abstract] OR "nasal allergy"[Title/Abstract]) AND ("nasal mucosa"[Title/Abstract] OR "nasal epithelium"[Title/Abstract] OR "nasal epithelial"[Title/Abstract] OR "nasal allergen challenge"[Title/Abstract] OR "nasal lining fluid"[Title/Abstract] OR "nasal biopsy"[Title/Abstract] OR "nasal biopsies"[Title/Abstract] OR "nasal brushing"[Title/Abstract] OR "nasal lavage"[Title/Abstract]) AND ("single-cell"[Title/Abstract] OR "single cell"[Title/Abstract] OR "spatial transcriptomics"[Title/Abstract] OR fibroblast*[Title/Abstract]) AND ("2016/01/01"[Date - Publication] : "2026/09/18"[Date - Publication])

## Barrier and repair — 164 records

("Rhinitis, Allergic"[Mesh] OR "allergic rhinitis"[Title/Abstract] OR "nasal allergy"[Title/Abstract]) AND ("nasal mucosa"[Title/Abstract] OR "nasal epithelium"[Title/Abstract] OR "nasal epithelial"[Title/Abstract] OR "nasal allergen challenge"[Title/Abstract] OR "nasal lining fluid"[Title/Abstract] OR "nasal biopsy"[Title/Abstract] OR "nasal biopsies"[Title/Abstract] OR "nasal brushing"[Title/Abstract] OR "nasal lavage"[Title/Abstract]) AND (barrier[Title/Abstract] OR repair[Title/Abstract] OR "tight junction"[Title/Abstract] OR S100A8[Title/Abstract]) AND ("2016/01/01"[Date - Publication] : "2026/09/18"[Date - Publication])

## Challenge and timing — 337 records

("Rhinitis, Allergic"[Mesh] OR "allergic rhinitis"[Title/Abstract] OR "nasal allergy"[Title/Abstract]) AND ("nasal mucosa"[Title/Abstract] OR "nasal epithelium"[Title/Abstract] OR "nasal epithelial"[Title/Abstract] OR "nasal allergen challenge"[Title/Abstract] OR "nasal lining fluid"[Title/Abstract] OR "nasal biopsy"[Title/Abstract] OR "nasal biopsies"[Title/Abstract] OR "nasal brushing"[Title/Abstract] OR "nasal lavage"[Title/Abstract]) AND ("late phase"[Title/Abstract] OR "late-phase"[Title/Abstract] OR "IL-13"[Title/Abstract] OR "IL-5"[Title/Abstract] OR priming[Title/Abstract]) AND ("2016/01/01"[Date - Publication] : "2026/09/18"[Date - Publication])

## Local effector and memory — 186 records

("Rhinitis, Allergic"[Mesh] OR "allergic rhinitis"[Title/Abstract] OR "nasal allergy"[Title/Abstract]) AND ("nasal mucosa"[Title/Abstract] OR "nasal epithelium"[Title/Abstract] OR "nasal epithelial"[Title/Abstract] OR "nasal allergen challenge"[Title/Abstract] OR "nasal lining fluid"[Title/Abstract] OR "nasal biopsy"[Title/Abstract] OR "nasal biopsies"[Title/Abstract] OR "nasal brushing"[Title/Abstract] OR "nasal lavage"[Title/Abstract]) AND ("local IgE"[Title/Abstract] OR "local allergic rhinitis"[Title/Abstract] OR FcεRI[Title/Abstract] OR omalizumab[Title/Abstract] OR plasmablast*[Title/Abstract] OR "B cell"[Title/Abstract] OR "T cell"[Title/Abstract] OR ILC2[Title/Abstract]) AND ("2016/01/01"[Date - Publication] : "2026/09/18"[Date - Publication])

## Physiology and translation — 388 records

("Rhinitis, Allergic"[Mesh] OR "allergic rhinitis"[Title/Abstract] OR "nasal allergy"[Title/Abstract]) AND ("nasal mucosa"[Title/Abstract] OR "nasal epithelium"[Title/Abstract] OR "nasal epithelial"[Title/Abstract] OR "nasal allergen challenge"[Title/Abstract] OR "nasal lining fluid"[Title/Abstract] OR "nasal biopsy"[Title/Abstract] OR "nasal biopsies"[Title/Abstract] OR "nasal brushing"[Title/Abstract] OR "nasal lavage"[Title/Abstract]) AND (neuroimmune[Title/Abstract] OR nerve*[Title/Abstract] OR secretion[Title/Abstract] OR exudation[Title/Abstract] OR biomarker*[Title/Abstract] OR endotype*[Title/Abstract] OR immunotherapy[Title/Abstract]) AND ("2016/01/01"[Date - Publication] : "2026/09/18"[Date - Publication])

# Exact executed Embase strategy

('allergic rhinitis'/exp OR 'allergic rhinitis':ti,ab,kw OR 'nasal allergy':ti,ab,kw) AND ('nasal mucosa':ti,ab,kw OR 'nasal epithelium':ti,ab,kw OR 'nasal epithelial':ti,ab,kw OR 'nasal allergen challenge':ti,ab,kw OR 'nasal lining fluid':ti,ab,kw OR 'nasal biopsy':ti,ab,kw OR 'nasal biopsies':ti,ab,kw OR 'nasal brushing':ti,ab,kw OR 'nasal lavage':ti,ab,kw) AND [2016-2026]/py AND ('single-cell':ti,ab,kw OR 'single cell':ti,ab,kw OR 'spatial transcriptomics':ti,ab,kw OR fibroblast*:ti,ab,kw OR barrier:ti,ab,kw OR repair:ti,ab,kw OR 'tight junction':ti,ab,kw OR s100a8:ti,ab,kw OR 'late phase':ti,ab,kw OR 'late-phase':ti,ab,kw OR 'il-13':ti,ab,kw OR 'il-5':ti,ab,kw OR priming:ti,ab,kw OR 'local ige':ti,ab,kw OR 'local allergic rhinitis':ti,ab,kw OR 'fcεri':ti,ab,kw OR fceri:ti,ab,kw OR omalizumab:ti,ab,kw OR plasmablast*:ti,ab,kw OR 'b cell':ti,ab,kw OR 't cell':ti,ab,kw OR ilc2:ti,ab,kw OR neuroimmune:ti,ab,kw OR nerve*:ti,ab,kw OR secretion:ti,ab,kw OR exudation:ti,ab,kw OR biomarker*:ti,ab,kw OR endotype*:ti,ab,kw OR immunotherapy:ti,ab,kw)

# Exact executed Web of Science strategy

TS=("allergic rhinitis" OR "nasal allergy" OR "hay fever" OR hayfever) AND TS=("nasal mucosa" OR "nasal epithelium" OR "nasal epithelial" OR "nasal allergen challenge" OR "nasal lining fluid" OR "nasal biopsy" OR "nasal biopsies" OR "nasal brushing" OR "nasal lavage") AND TS=("single-cell" OR "single cell" OR "spatial transcriptomics" OR fibroblast* OR barrier OR repair OR "tight junction" OR S100A8 OR "late phase" OR "late-phase" OR "IL-13" OR "IL-5" OR priming OR "local IgE" OR "local allergic rhinitis" OR "FcεRI" OR FCERI OR omalizumab OR plasmablast* OR "B cell" OR "T cell" OR ILC2 OR neuroimmune OR nerve* OR secretion OR exudation OR biomarker* OR endotype* OR immunotherapy) AND PY=(2016-2026)

# Source verification and remaining limitations

The original 63 references were resolved against PubMed by DOI. New references 15, 52, 61, 69, and 71–74 were checked against individual PubMed records after identification in the supplemental retrievals; this verification does not retroactively place them in the executed 823-record PubMed set. Full-text focused checks confirmed the surgical cohort and six-sample explant assays for reference 52 and the exposure protocols for reference 73. Other added summaries use verified indexed abstracts; assay-level denominators must not be extrapolated from total enrollment. S1 now contains 36 representative publications. “Not reported” indicates that the requested information was not established in the accessible primary record; it does not imply that the information is absent from the complete study documentation. Full text and supplementary files for reference 7 confirm 49 participants overall: scRNA-seq included 25 AR and 14 NAR, scATAC-seq used a 16/8 subset, and an additional immunofluorescence cohort comprised 5/5. Sampling season, current allergen-exposure phase, exact anatomical subsite, and intranasal-corticosteroid/antihistamine washout were not reported. Reference 8 retains the unresolved internal discrepancy between the abstract/results (10/group) and participant-recruitment methods (9/group), together with its nasal-polyposis context. Database vocabulary, institutional edition access, update date/year differences, the chosen recent window, accessible texts and purposive appraisal limit coverage. Broader retrieval does not eliminate narrative selection bias.

# Retrieval audit

Supplementary Retrieval Audit S4 consolidates the database-level counts, executed-query summary, report-level reconciliation and the 25-record focused candidate-appraisal log. Valid original exports are retained by the authors and are available on request. Bibliographic and registry records remain distinct. S4 documents retrieval and focused appraisal; it does not establish that every retrieved record was eligible or fully read.
